# Developing monitoring and evaluation tools for event-based surveillance: experience from Vietnam

**DOI:** 10.1186/s12992-020-00567-2

**Published:** 2020-04-30

**Authors:** Alexey Clara, Anh T. P. Dao, Anthony W. Mounts, Christina Bernadotte, Huyen T. Nguyen, Quy M. Tran, Quang D. Tran, Tan Q. Dang, Sharifa Merali, S. Arunmozhi Balajee, Trang T. Do

**Affiliations:** 1 Division of Viral Diseases, National Center for Immunization and Respiratory Diseases, Centers for Disease Control and Prevention, Atlanta, GA USA; 2 Division of Global Health Protection, Center for Global Health, Centers for Disease Control and Prevention, Atlanta, GA USA; 3 PATH, Seattle, WA USA; 4 PATH, Hanoi, Vietnam; 5 General Department of Preventive Medicine, under the Vietnam Ministry of Health, Hanoi, Vietnam

**Keywords:** Event-based surveillance, Monitoring and evaluation tools, Vietnam

## Abstract

**Background:**

In 2016–2017, Vietnam’s Ministry of Health (MoH) implemented an event-based surveillance (EBS) pilot project in six provinces as part of Global Health Security Agenda (GHSA) efforts. This manuscript describes development and design of tools for monitoring and evaluation (M&E) of EBS in Vietnam.

**Methods:**

A strategic EBS framework was developed based on the EBS implementation pilot project’s goals and objectives. The main process and outcome components were identified and included input, activities, outputs, and outcome indicators. M&E tools were developed to collect quantitative and qualitative data. The tools included a supervisory checklist, a desk review tool, a key informant interview guide, a focus group discussion guide, a timeliness form, and an online acceptability survey. An evaluation team conducted field visits for assessment of EBS 5–9 months after implementation.

**Results:**

The quantitative data collected provided evidence on the number and type of events that were being reported, the timeliness of the system, and the event-to-signal ratio. The qualitative and subjective data collected helped to increase understanding of the system’s field utility and acceptance by field staff, reasons for non-compliance with established guidelines, and other factors influencing implementation.

**Conclusions:**

The use of M&E tools for the EBS pilot project in Vietnam provided data on signals and events reported, timeliness of reporting and response, perceptions and opinions of implementers, and fidelity of EBS implementation. These data were valuable for Vietnam’s MoH to understand the function of the EBS program, and the success and challenges of implementing this project in Vietnam.

## Background

All member states of the World Health Organization (WHO) are required by the International Health Regulations (IHR, 2005) to have an early warning and response (EWAR) system [1]. In order to support the implementation of the IHR, the Global Health Security Agenda (GHSA) was launched in February 2014. GHSA strengthens both global capacity and nations’ capacity to prevent, detect, and respond to infectious threats [2, 3].

IHR (2005) emphasize development of capacities for collecting and analyzing information from sources outside the health system itself [1, 4], including detection of unusual events that may represent emerging threats. Event-based surveillance (EBS) is a component of EWAR that can significantly improve the sensitivity of a system to detect emerging outbreaks [5, 6]; effective monitoring and evaluation (M&E) of an EBS system can help ensure consistent system performance. However, a limited number of EBS M&E tools have previously been available in the published literature [7–13].

Monitoring in the context of surveillance refers to routine and ongoing tracking of (a) implementation of planned surveillance activities, (b) data gathered by the surveillance system, and (c) overall performance of the system. Evaluation of a surveillance system is periodic assessment of the relevance, effectiveness, and impact of activities of surveillance, and focuses on whether the system meets its objectives and makes effective use of resources [5, 6, 14, 15]. Routine monitoring of system performance and periodic wider system evaluations should be conducted as part of any surveillance system [5, 14]. When correctly implemented, M&E can promote the best use of data collection resources, ensure that the surveillance system meets its intended objectives, provide signs of potential system deviations, and identify opportunities for performance improvements [5, 6, 14, 15].

As part of GHSA program implementation, Vietnam’s MoH launched a two-phase project to implement EBS in six provinces in collaboration with the US Centers for Disease Control and Prevention (CDC) and the non-governmental organization PATH in September 2016 [16, 17]. A package of M&E tools was developed and deployed for this EBS project. Data on the process of implementation, deployment of the M&E tools, and results of the implementation showed EBS resulted in early detection and reporting outbreaks, improved collaboration between the healthcare facilities and preventive sectors of the ministry, and increased community participation in surveillance and reporting. In addition, the pilot demonstrated the value of supportive supervision and evaluation [16, 17]. This manuscript describes the development and content of the M&E tools used in the EBS project, and how the M&E strategy was integrated within EBS implementation.

## Methods

### Implementation of the EBS pilot project

Vietnam has 4 administrative health regions (north, south, central coast, and central highland); each has a regional public health institute (RI) that is responsible for the overall technical direction and supervision of surveillance and response to diseases and outbreaks in that region [17]. Within each region, provincial preventive medicine centers (PPMCs) lead surveillance and response activities within their respective provinces. Within a province, the district health centers (DHCs) coordinate public health activities in each of the districts. Districts are divided into communes, and each commune has a commune health station (CHS). The CHS is the primary healthcare unit in Vietnam [18] and is usually staffed by a physician, a nurse, and a midwife. Within each commune, village health workers (VHWs; rural areas) and health collaborators (HCs; urban areas) constitute community networks and support the CHSs in different health-promotion activities. VHWs and HCs were trained through the current project to function as key informants, collecting information from the community and reporting as needed to the CHS. For the pilot project, the CHSs sensitized VHWs and HC to detect and report signals. Besides VHWs and HCs, some additional community members such as community leaders, school teachers, pharmacy workers, and veterinarians were also invited to participate in the EBS pilot and received training to recognize and report signals.

The MoH launched EBS implementation in 2 phases. Phase 1 started in September 2016 in 4 provinces (Quang Ninh and Nam Dinh in the north region, and An Giang and Ba Ria Vung Tau in the south region). Phase 2 started in August 2017 with addition of two more provinces (Dak Nong in the central highlands region and Binh Thuan in the central coast region).

To support the implementation of the EBS pilot, the MoH’s General Department of Preventive Medicine (GDPM) formed an EBS Technical Working Group (TWG) with experts from the MoH (including the 4 RIs), CDC, PATH, WHO, and technical staff from the PPMCs of the participating provinces [16]. The TWG drafted a list of signals to be used at community and health facility levels (Table 1). The list was based on prioritization criteria that included diseases that have a great impact on public health and significant epidemic potential, emerging or reemerging diseases, and diseases that are scheduled for eradication or elimination. The list of signals provided guidance for signal detection at community level and health facilities. The TWG also drafted interim technical guidelines, standardized operative procedures, and M&E tools that were used to launch the project.

**Table 1:** List of signals for community level and health facilities

For community level	For health facilities
1. A child less than 15 years old with sudden weakness of limbs	1. Healthcare workers with severe illness requiring hospital admission or resulting in death, after caring for patients with similar symptoms. 2. Two or more cases of severe acute respiratory infections within 7 days in the same community, household, school or workplace. 3. One case of severe viral pneumonia requiring hospital admission. 4. Unexpectedly large increase of cases of the same symptoms, based on clinician’s professional judgements. 5. Two or more cases of infectious diseases with the same symptoms from the same location (e.g. household, residential unit, school, factory, etc.). 6. One case of malaria in an area where the disease has been eliminated or never circulated before. 7. Occurrence of unexplained or unusual clinical manifestation or treatment response of a known infectious disease based on clinician’s professional judgements. 8. Occurrence of one or more cases or deaths of a strange, unusual or unexplained disease, based on clinician’s professional judgments. 9. Unexpected increase of people being vaccinated for rabies in the same community. 10. Any suspected cases of communicable diseases of group A according to the Law on Prevention and Control of Infectious Diseases (2007).
2. A single case with fever and rash, accompanied by cough or pink eyes	
3. A single case that is severe enough to require hospital admission or dies with any of the followings: a) Three or more rice watery stools within 24 hours in any person 5 years old or older with dehydration. b) Respiratory infection with fever in someone who has been traveling abroad in the last 14 days. c) Respiratory infection with fever after contact with live poultry in the last 14 days. d) Illness within 7 days following vaccination. e) Illness which has never been seen before, or with rare symptoms, in the community. f) An unexplained death.	
4. Two or more hospitalized cases and/or death(s) with similar symptoms occurring in the same community, school, or workplace within 7 days.	
5. Unusual large numbers of one of the followings: a) Children absent from the same school due to the same illness within 7 days. b) People buying medicines for fever, cough, or diarrhea at pharmacies in the same residential area within 1 week. c) People sick with similar types of symptoms at the same time. d) Sickness or die-off of poultry, domestic animals or other animals.	
6. Any dog that: a) Is suspected as a rabid dog b) Is sick and has bitten someone c) Has bitten two or more people in the last 10 days.	

Thirty-three master trainers from the national, regional, and provincial level were trained on EBS. After this, all public health system staff in participating provinces were trained by the cascade training method, including VHWs/HCs, CHS staff, DHC staff, and hospital healthcare workers. Implementation of the project began when the trainings were completed. EBS implementation in each province was the responsibility of the surveillance staff at each level. An EBS focal point was designated at each level to coordinate project activities.

**Fig. 1:**
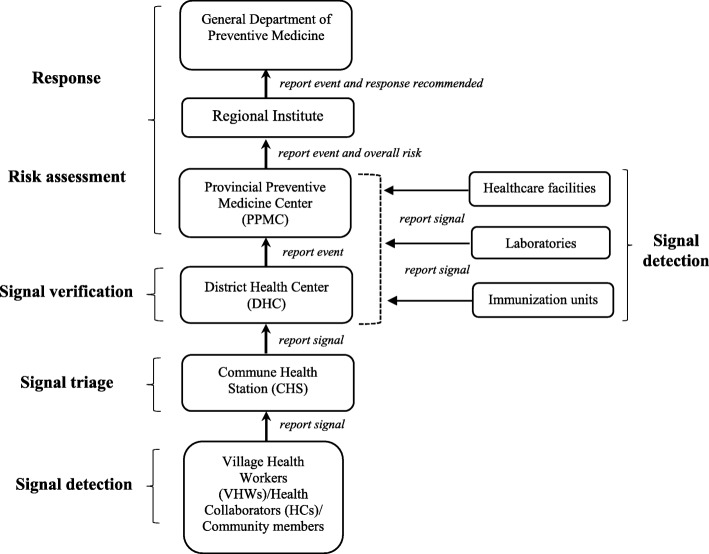
Existing surveillance and reporting system improved for the event-based surveillance pilot project in Vietnam. Boxes represent different health system levels participating in event-based surveillance and arrows indicate flow of information from the community and health facilities to the national level. Event-based surveillance steps are marked in bold

Signals detected or received by VHWs/HCs were reported immediately to the EBS focal point at CHSs, by phone calls or in person. Upon notification of signals to the CHS, the corresponding EBS focal point triaged the signals to screen out data/information that were not relevant for early detection purposes. Once a signal was triaged, it was reported to the DHC by phone/email and the district EBS focal point verified if the signal represented a real public health threat. The purpose of the signal verification was to determine whether the reported event truly occurred and collect information on the characteristics of the event. Once a signal was verified, it was referred to as an event. Signals detected by healthcare workers at health facilities were reported immediately to the corresponding PPMC or DHC’s EBS focal point, who conducted triage and verification. All events were reported to the PPMC, which assigned the level of risk and impact of each event through a risk assessment. The risk assessment determined the type of response to be initiated by the public health system [16, 17] (Fig. 1). Logbooks and monthly summary reports forms were used to record signals and events data at all levels.

### Design and development of M&E tools

**Fig. 2:**
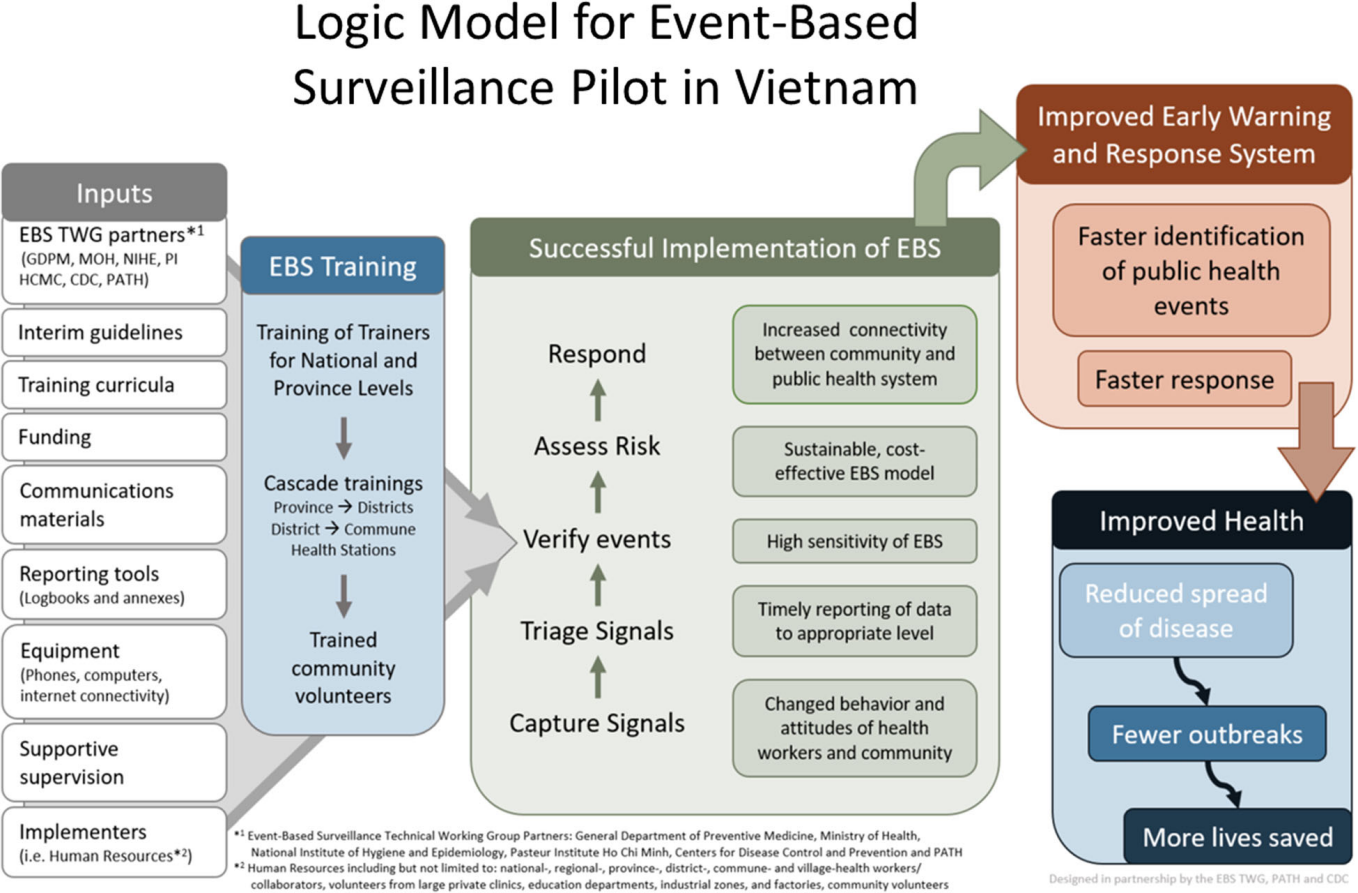
Logic model for event-based surveillance pilot in Vietnam. This figure shows the main components of EBS implementation pilot and how inputs, activities, and outputs are articulated in a strategic EBS framework to achieve intended outcomes and impact on population health

The TWG developed a strategic EBS framework (logic model) for the country based on the EBS implementation pilot project’s goals and objectives (Fig. 2). This conceptual map identified main components of the EBS implementation pilot and demonstrated how they relate to one another. The EBS logic model included ongoing monitoring and a detailed assessment of the entire system 5–9 months after EBS implementation using tools developed for those purposes.

Input, output, and outcome indicators were developed to reflect the goals of the EBS framework (Table 2). Input indicators referred to the resources needed to implement EBS, while output indicators were measures of the immediate results of EBS-related activities. Outcome indicators measured the quality of the surveillance system and the extent to which surveillance objectives were achieved.

**Table 2:** Input, outputs and outcomes measures for event-based surveillance in Vietnam

**Main components**	**Inputs**	**Activities**	**Outputs**	**Outcomes**
	1. Provision of resources 2. Formation of TWG 3. List of signals 4. Materials/equipment *a. Technical guidelines* *b. Training materials* *c. Reporting tools* *d. Communication materials* *e. Computers, phone credits for VHWs* 5. Technical assistance	1.1.1. Stakeholder meetings to draft signals and plan implementation 2. Completion of: *a. Technical guidelines* *b. Training materials* *c. Reporting tools* *d. Communication materials* 3. Trainings *a. Training of Trainers* *b. Cascade trainings* 4. EBS FPs identified 5. Distribution of materials/equipment 6. Monitoring visits	1.1.1. List of signals available 2. Materials completed 3. Trainings conducted as planned 4. EBS FPs designated 5. Materials/equipment distributed to all levels 6. Monitoring visits conducted by provinces	**Short-term outcomes:** 1. Signals detected and reported 2. Events reported and responded
				**Intermediate outcomes:** 1. Time of notification and response 2. High acceptance of EBS by implementers at each level 3. Increased trust among the community
				**Long-term outcomes:** 1. Reduction of morbidity and mortality associated with infectious diseases
**Questions**	**Process evaluation questions**			**Outcomes evaluation questions**
	1. Were all necessary resources available to implement EBS at each level? 2. Were EBS training and monitoring visits carried out as planned at each level? 3. Were the trainings effective? 4. What were the barriers and facilitators identified by EBS implementers at each level?			1. How many signals and events were reported and responded? 2. Were events reported and responded to in < 48 h? 3. What is the acceptance of EBS? 4. What is the understanding of EBS?
**Indicators**	**Input indicators**^a^	**Output indicators**^a^		**Outcome indicators**
	1. TWG created and functional 2. Materials and equipment available *a. Technical guide* *b. Training* *c. Reporting tools* *d. Communication* *e. Computers, phone credits for VHWs*	1. Number of EBS focal points assigned at each level 2. Number of notebooks distributed to VHWs 3. Number of log books distributed to EBS focal points at district level 4. Number of log books distributed to EBS focal points at commune health stations 5. Number of EBS poster distributed to commune health stations and hospitals 6. Number of EBS flyers/brochures distributed to commune health stations and hospitals 7. Number of training of trainers conducted by GDPM and regional institutes 8. Number of cascade trainings conducted at each level 9. Number of assigned EBS focal point trained in EBS in each province 10. Number of village health workers trained in EBS in each province 11. Number and percentage of districts implementing EBS in each province 12. Number and percentage of public hospitals implementing EBS in each province 13. Number and percentage of communes implementing EBS in each province 14. Number of monitoring visits conducted by each province since project was launched 15. Barriers and facilitators identified by EBS implementers at each level 16. Fidelity of EBS technical guidelines implementation at each level		*a) Signals and events reported*^*b*^ 1. Number of signals reported 2. Number of signals triaged 3. Number of signals verified 4. Number of events reported 5. Number of events assessed 6. Number of events responded to *b) Timeliness of reporting/response*^a^ 7. Time in hours from signal detection to event reporting to the district level 8. Time in hours from signal detection to the response *c) Sensitivity and specificity*^a^ 9. Signals appropriately sensitive and specific to detect real events *d) Acceptance of EBS*^c^ 10. % of survey respondents at each level who agreed that EBS is very important in the detection of events 11. % of survey respondents at each level who agreed that EBS helps detect events earlier than before implementation 12. % of survey respondents at each level who were willing to continue taking part in EBS 13. % of survey respondents at each level who agreed that EBS should be continued
**Tools**	**For monitoring:** Supervisory checklist		**For evaluation:** Desk review tool; key informant interview guide; focus group discussion guide; timeliness form; online acceptability survey	

*TWG* technical working group, *VHWs* village health workers, *EBS* event-based surveillance, *EBS FPs* event-based surveillance focal points, *GDPM* The General Department of Preventive Medicine (of Vietnam)

^a^over 9 calendar months for Phase 1 provinces and over 5 calendar months for Phase 2 provinces

^b^one calendar month per province

^c^In June–July 2017 per province (phase 1), and in January 2018 per province (phase 2)

### Using the monitoring tools

As a basic tool for data monitoring, each administrative unit within a province completed a monthly summary report of signals and events and sent the report via email to EBS focal points at the next level. Each of the regional-level EBS focal points compiled the data from the provinces and shared them with the national-level EBS focal points. Personnel at the regional level were responsible for reviewing these data and liaising with the provincial level EBS focal points, who in turn coordinated with the EBS focal points at the district and commune levels as needed. In addition, a supervisory checklist was developed for each administrative level (provincial, district, and commune) to be used during routine monthly or every-other-month supportive monitoring visits during the pilot implementation phase. EBS focal points involved in monitoring were trained before monitoring tool implementation.

Supportive supervisory visits were conducted by EBS focal points at each administrative level, with staff from higher levels visiting staff at lower levels. The visits were intended to identify and correct problems during implementation and for provision of technical assistance, mentoring, and hands-on refresher training as needed. The supervisory checklist was structured to collect data from direct observations, documents such as log books, and interviews, and allowed supervisors to evaluate implementation fidelity as well as understand personal perceptions and opinions of the implementers regarding the program.

The checklist covered five main areas of implementation: EBS staffing, training, availability of resources needed to implement EBS (equipment, guidelines, forms, etc.), whether or not monitoring visits were being made to lower administrative levels, and problems with filling in records and forms (Table 3).

**Table 3:** Deployment of evaluation tools and target population per administrative level

Field visits					
Level	Desk review	Key informant interview	Focus group discussion	Timeliness form	Acceptability survey
**Province** *(Field visits were conducted to all pilot provinces)*	• Target: - The PPMC’s EBS focal point/EBS team in each pilot province	• Target: - The PPMC’s EBS focal point in each pilot province - The province hospital’s EBS focal point in each pilot province	• Target: - DHC’s EBS focal points of selected districts in each province	None	• Target: - An online survey was open to all PPMC’s EBS focal points in Phase 1 provinces
**District** *(Field visits were conducted to 2 selected districts in each pilot province)*	None	• Target: - The DHC’s EBS focal point in each selected district - The district hospital’s EBS focal point in each selected district	None	• Target: - The DHC’s EBS focal point of each district in pilot provinces	• Target: - An online survey was open to all DHC’s EBS focal points in Phase 1 provinces
**Commune** *(Field visits were conducted to 2 selected communes [CHS] in each selected district)*	None	• Target: - The CHS’s EBS focal point in each selected district	• Target: - VHWs from selected communes in each selected district • Community members in each selected commune	None	• Target: - An online survey was open to all CHS’s EBS focal points and all VHWs in Phase 1 provinces

### Using the evaluation tools

An evaluation team was formed consisting of stakeholders from GDPM, RIs, CDC, and PATH and designed five data collection tools for assessment of EBS 5–9 months after implementation to document products of EBS activities, perceptions of implementers, and fidelity of implementation. The tools included a desk review tool, a key informant interview guide, a focus group discussion guide, a timeliness form, and an online acceptability survey (applied in 4 Phase 1 provinces only). The main process and outcome components were identified and included input, activities, outputs, and short-term, intermediate-term, and long-term outcomes. Thirty-one indicators were developed for M&E purposes based on the EBS framework (Table 2). Field visits to all pilot provinces and selected districts/communes were scheduled (2 districts per province and 2 communes per selected district). In addition, target populations were identified for each evaluation tool at each administrative level (Table 3). Evaluation team members were trained how to use evaluation tools before their implementation.

The desk review tool collected data on number of EBS trainings and trainees; number and percentage of districts, communes, and hospitals implementing EBS; number of communications and registering materials (posters, brochures/flyers, and notebooks) delivered to local levels; number of signals and events reported from lower to upper levels each month; completeness of logbooks; and monthly allowance/incentives for implementers in each province (Table 4). The desk review tool was sent to participating provinces in advance of visits, allowing them 2 weeks prior to the field evaluation activities to complete it, and EBS staff reported that the time was sufficient to review primary sources of information (such as the commune-level log books of EBS signals) and compile accurate data.

**Table 4:** Description of monitoring and evaluation tools for event-based surveillance in Vietnam

	Tool	Content	Data collection methods
**Monitoring**	Supervisory checklist	1. EBS staffing 2. Training in EBS 3. Availability of materials and equipment 4. Monitoring visits 5. Revision of records/forms (e.g., logbooks, verification forms)	• Monitoring visits at each level • Interviews with EBS focal points in each province, district, and community health station • Document review
**Evaluation**	Desk review tool	1. Training in EBS 2. System coverage 3. Materials 4. Monthly summary reports 5. Review of proper use of log books 6. Monthly allowance and incentives for implementers	• Provincial EBS focal points completed the tool prior to evaluation site visits • Evaluation visits at province level • Interviews with EBS focal points at province level • Document review
	Key informant interview guide^a^	1. Fidelity of EBS implementation 2. Timeliness 3. Perceived value and acceptance of EBS 4. Costs 5. Lessons for future roll-ou	• Evaluation visits to provinces and select districts, hospitals, and community health stations • Interviews with EBS focal points at all levels
	Focus group discussion guide^b^	1. Fidelity to EBS guidelines implementation 2. Timeliness 3. Costs 4. Perceived value and acceptance of EBS 5. Reporting to the electronic surveillance system 6. Lessons for future roll-out	• Evaluation visits to provinces and select districts, hospitals and community health stations • Focus group discussions conducted with EBS focal points in select districts • Focus group discussions conducted with village health workers and key informants in select communitie
	Timeliness form	1. Type of event 2. Date and time of signal onset 3. Date and time when signal were registered in the commune health station logbook 4. Date and time when event were registered at the district level 5. Date and time when a response for the event was initiated 6. Response activities implemented	• Timeliness form was sent via email to all districts in provinces; EBS focal points in each district completed the form
	Online acceptability survey	1. Demographic profile of respondents 2. Personal beliefs, values, and attitudes toward EBS 3. Possible barriers to participation in EBS 4. Active informants in the community [for commune health stations] 5. Facilitating factors to implement EBS 6. Government support	• Electronic survey was available for online entry data • EBS focal points at all levels and village health workers in all participating communities were invited to complete the survey

^a^There was a shortened version for EBS focal points at health facilities that included fidelity to EBS implementation, perceived value of EBS/acceptability, and questions about lessons learned that were applicable to future roll-out

^b^Key informant in the community version included current knowledge of EBS, reporting, and perceived value of EBS/acceptability

The key informant interview tool consisted of semi-structured in-depth interviews with EBS focal points to obtain qualitative information on fidelity of EBS implementation, timeliness of reporting and response to events, perceived value and acceptance of EBS, and lessons learned that were applicable for future roll-out. A shortened version of the tool for interviews with hospital EBS focal points was developed and focused on fidelity of EBS implementation, perceived value and acceptance of EBS, and lessons learned (Table 4).

The focus group discussion tool collected qualitative data about EBS implementation at the district and commune levels. Different guides were developed for three specific target groups: (a) EBS focal points at the district level (for both clinical and preventive medicine sectors), (b) VHWs and HCs, and (c) community members serving as key informants, such as schoolteachers and pharmacy workers or members of social unions/associations. The tool collected data on fidelity to EBS interim guidelines implementation, timeliness of reporting and response to events, time spent working in EBS, perceived value and acceptance of EBS, reporting of cases of infectious diseases to the electronic communicable disease surveillance system, and lessons for future roll-out. The questions in the focus group discussion also sought to increase understanding of how well community members understood the signals, how the community health workers detected signals in the community, which strategies engaged community members to participate in EBS, and the perceived value and acceptance of EBS (Table 4).

The timeliness form collected data including the type of the event, date and time of signal onset (i.e., when the signal appeared), time of signal registration (i.e., when the signal was first registered in the corresponding logbooks), time of event confirmation (i.e., when the event was registered at the district level), time of response (i.e., when a response for the event was initiated), and the type of response implemented. The timeliness form was sent electronically to the EBS focal point at each DHC in the pilot provinces, who collected data and returned the completed form to evaluation team.

The online acceptability survey included questions on demography; personal beliefs, values, and attitudes toward EBS; possible barriers to participation in EBS; identity and roles of active informants in the community; facilitating factors for EBS implementation; and specific forms of government support. Most survey questions were based on a Likert scale model. Additional open-ended questions asked respondents to provide recommendations to improve EBS (Table 4). From June to July 2017, the survey was open to all VHWs/HCs, CHSs, DHCs, and PPMCs of Phase 1 participating provinces. The survey was not open for the Phase 2 pilot provinces.

The evaluation tools were developed using Microsoft Word 10.0. and hard copies were printed for site visits. The acceptability survey was uploaded to the ONA platform (Ona Systems, Inc.) for online entry data. Quantitative and qualitative data were stored in Microsoft Excel 2010.

## Results

The checklist facilitated the identification of deviations from the intended implementation process, and the prompt provision of practical recommendations to solve issues on the spot. Clara et al. showed how the event-to-signal ratio, calculated as events detected per month divided by signals detected per month from September 2016 to December 2017 in the 6 pilot provinces, increased at specific points that roughly corresponded to specific interactions that included supportive M&E visits [17]. As Vietnam is in the process of scaling up EBS nationwide, and with EBS becoming an integral part of routine surveillance system, EBS supervisory visits will be integrated into existing routine supervisory visits for other public health purposes (e.g., supervision of nutrition programs, family planning, or general surveillance programs).

**Fig. 3:**
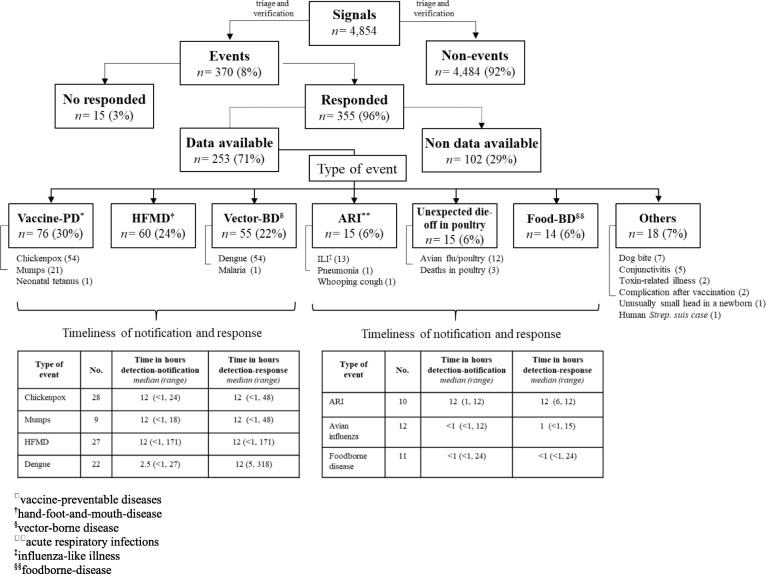
Signals, type of events reported, and timeliness of notification and response for the event-based surveillance pilot in Vietnam. The flowchart gives an overview the number of signals and events that were reported during September 2016 to December 2017 by the 6 pilot provinces as well as how many events were responded and type of events when data were available. Type of events were organized in 6 categories and breakdowns of specific events have been added under each category. Tables at the bottom showed the median time and range in hours from detection to notification and from detection to response by 7 specific type of events. Tables included the number of events with timeliness data available

Desk review showed that a total of 8661 VHWs/HCs, 1379 CHS staff, 185 DHC staff, and 75 hospital healthcare workers were trained on EBS. Pilot provinces reported 4854 signals and 370 (8%) events from September 2016 to December 2017 [16, 17]. Information on type of event was available for 253 events and included a variety of vaccine-preventable diseases (e.g., chickenpox and mumps), zoonoses such as avian influenza in poultry, vector-bone diseases (e.g., dengue and malaria), foodborne disease outbreaks, and other non-infectious conditions (e.g., toxic-related illness and complication after vaccination) (Fig. 3). A response was implemented in 355 events (96%).

In total, 51 key informant interviews and 46 focus group discussions were conducted during field visits. The most common challenges that interviewees mentioned about signal detection were: a) need for refresher trainings for VHWs/HCs, b) VHWs’/HCs’ inability to cover large geographic areas, and c) limited implementation in urban areas. Suggestions included a need for ongoing supervision/refresher trainings to continue in order to identify and resolve similar problems; expansion of the network of information sources in the community to improve detection; and the need to refine some signals in the community and hospitals to facilitate their understanding. All interviewees stated they did not encounter major challenges when reporting signals/events to upper levels.

Conducting both evaluation tools (focus group discussions and key informant interviews) for different target groups enabled the collection of qualitative data including personal beliefs, values, and attitudes toward EBS. They were effective in documenting the perceived accuracy and the observed accuracy with which the five EBS steps (detection, triage, verification, risk assessment, and response) were carried out. Additionally, the interviews provided an opportunity for the EBS focal points at the province and district levels to share their views on successes and challenges of EBS. The interviewees presented many case studies that demonstrated the importance of EBS to their work around the surveillance of and response to infectious diseases and outbreaks.

Information on timeliness of reporting and response to events were available for 210 (57%) of 370 events. Timeliness data showed that the median times in hours from detection to notification and detection to response were within 24 h and 48 h, respectively. Avian influenza in poultry events and foodborne disease outbreaks showed the shortest median time from detection to notification and response (1 h or less) while [16, 17] (Fig. 3).

Although the timeliness form was simple, with only six requested variables to collect, its completion presented challenges for two main reasons: (1) it required compiling data that had to be acquired from logbooks in different physical locations and different levels of the health system and (2) some data elements required retrospective data collection, such as the date of signal occurrence, date of confirmation of events, etc. An electronic reporting system would have greatly facilitated collection of these data. At the time, the paper records lacked accurate date and time stamps.

The online acceptability survey was a cost-effective way to reach a large number of implementers at all levels in a relatively short period. A total of 1633 (22.8%) of the 7167 VHWs, 428/653 CHS EBS focal points (65.5%), 39/43 DHC EBS focal points (91%) and all PPMC EBS focal points from the 4 Phase 1 provinces completed the online acceptability survey [17]. Percentages of respondents from all administrative levels who agreed/strongly agreed that EBS is very important in detection of events, helps detect events earlier, and should be continued ranged from 80 to 88%. Based on survey results, 86% of VHWs/HCs and CHS EBS focal points, and 90% of DHC focal points, reported being willing to continue taking part in EBS.

The use of the Likert scale simplified the filling process: the answers were easily quantifiable and allowed the participants to respond in a degree of agreement. However, the format of Likert scale items may have resulted in responses being influenced by previous questions or heavily biased to a neutral response, avoiding the extremes [19, 20]. In addition, because it was a self-administered survey, participants may have had different interpretations of the questions and difficulties in understanding how to respond (e.g., how to rank potential barriers for EBS implementation). Finally, the VHWs/HCs who wanted to participate in the survey had to go to CHSs with an internet connection to access the survey online. This may have been a barrier for those interested in participating, and therefore contributed to lower response rates among VHWs/HCs compared to consistently higher response rates at higher levels of the health system (i.e., the provincial, district, and CHS levels).

## Discussion

Lack of reference models available for quantitative and qualitative measurements of EBS prompted the development of new tools for the pilot project (Additional file 1). The use of a variety of tools capable of extracting and triangulating both quantitative and qualitative data increased the ability to understand better the nuanced characteristics of the system. The quantitative data collected helped provide evidence on the number and type of events that were being reported, the timeliness of the system, and the event-to-signal ratio. However, it was not possible to estimate impact of EBS due to lack of baseline data before EBS implementation. The qualitative and subjective data collected helped to increase understanding of the system’s field utility and acceptance by field staff, associated human resource costs, reasons for non-compliance with established guidelines, and other factors influencing implementation that are important but often difficult to quantify.

Although a framework for short, intermediate, and long-term outcomes (Table 2) was designed early and provided a framework for implementation, it was difficult to measure some outcomes. For example, the outcome “increased trust among the community” was a challenging one, although the focus group discussions with VHWs and community members provided some understanding of their willingness to participate (or continue participating) in EBS. Data to estimate the long-term outcome “reduction of mortality associated with infectious diseases” were not available during the evaluation. It may be important to revise the outcome indicators and consider developing more feasible impact indicators for future studies.

It must be noted that, given the diversity and the large number of actors and levels involved in the evaluation, the collection of data using the tools involved the implementation of a complex and intense schedule of activities that was time and resource intensive. The collation and subsequent analysis of data collected during the focus group interviews of workers and key informants was especially challenging due to time-consuming activities such as recording, transcription, and translation of the interviews, and the proper interpretation of the findings.

With the information collected from the M&E process, the evaluation team recommended that the MoH Vietnam improve EBS data quality through: a) encouraging regular use of the verification form for all events; b) simplifying the monthly summary report form; c) ensuring each district records all events verified, including basic information such as type of event, and date/time of onset, detection, notification, and response; d) developing an electronic data management system for EBS reporting, and e) conducting refresher trainings on how to register and document signals and events properly. In addition, the TWG revised the EBS implementation guidelines and training materials before scale-up nationwide. The results of the evaluation and lessons learned were shared with MoH decision makers, and in March 2018, the MoH issued a mandate to incorporate the EBS program into Vietnam’s national surveillance platform.

The tools developed for the project have been customized and deployed in other countries including India, Cameroon, Ghana, and Kenya. All the tools are available in additional files, and countries implementing EBS are engaged to review these tools. Experience gained from implementing this project has guided the drafting of the Africa CDC framework for EBS [21]. It is hoped that these resources, if used appropriately, will help accelerate the effectiveness of EBS programs globally.

## Conclusion

The use of the M&E tools designed and developed for the EBS pilot project in Vietnam provided data on signals and events reported, timeliness of reporting and response, perceptions and opinions of implementers, and fidelity of EBS implementation. These data were valuable for the Vietnam’s MoH to understand the function of the EBS program, and the success and challenges of implementing this project in Vietnam. The EBS framework and the indicators and the tools developed for Vietnam can easily be customized to be used in any other country implementing EBS.

## Supplementary information

**Additional file 1.** Monitoring and evaluations tools for event-based surveillance in Vietnam. This file contains supervision checklists, desk review tool, key informant interview guides, focus group discussion guides, timeliness form, and acceptability surveys.

## Data Availability

Authors can confirm that all relevant data are included in the article and/or its supplementary information file.

## Abbreviations

CDC: US Centers for Disease Control and Prevention
DHC: District health center
CHS: Commune health station
EBS: Event-based surveillance
EWAR: Early warning and response
FP: Focal point
GDPM: General Department of Preventive Medicine
GHSA: Global Health Security Agenda
HC: Health collaborator
WHO: World Health Organization
M&E: Monitoring and evaluation
MoH: Ministry of Health
PPMC: Provincial preventive medicine center
TWG: EBS Technical Working Group
VHW: Village health workers
