# Supplementary material for: Developing monitoring and evaluation tools for event-based surveillance: experience from Vietnam
Source: Global Health. 2020 Apr 30;16:38. doi: 10.1186/s12992-020-00567-2 (PMC7191785; doi:10.1186/s12992-020-00567-2)
Supplement: Supplementary file 1 — Additional file 1. Monitoring and evaluations tools for event-based surveillance in Vietnam. This file contains supervision checklists, desk review tool, key informant interview guides, focus group discussion guides, timeliness form, and acceptability surveys. [file 12992_2020_567_MOESM1_ESM.pdf]

EBS Supervision Checklist-Commune Health Station (CHS)

**Name of CHS that is receiving the supportive supervision visit:**	
**Province and District where Commune Health Station is located:**	

This form should be used for visits to CHS

Date of visit: _________/_________/__________

(If no is answered for any of these questions, then please include a written explanation of why the answer was "no")

Date of last supportive supervision visit in this Commune Health Station: _____/_____/____

This form is designed for the use of regional/provincial/district supervisors to conduct supervision visits to the commune health station level.

**Name of lead supervisor:**		**Position of lead supervisor:**	
**Name of interviewee answering questions during this visit:**		**Position of interviewee:**	

**Questions to be asked during each supervision visit:**		Answer		Why not?	Recommended Actions
		Yes	No		
1	**Has CHS designated someone as the focal point person for EBS?**<br>If yes, Name:___________________________________<br>*(Ideally the focal point for EBS should be the person answering these questions)*				
2	**Has the staff responsible for EBS changed since the last supportive supervision visit?**<br>If yes, name of new EBS staff:				
	Has new staff received EBS training?				
	**Training:**				
3	**Have at least 2 people in this CHS received EBS training?**				
	If EBS staff is being interviewed, ask:<br>*Can you provide the names and contact information (phone, email) of $\geq$2 DHC/DPMC staff to whom signals can be reported?*<br>*Name & Email: ___________________________*<br>*Name & Email: ___________________________*				
	Were any refresher trainings attended by EBS staff from this CHS since the last visit?				

EBS Supervision Checklist-Commune Health Station (CHS)

**Name of CHS that is receiving the supportive supervision visit:**	

This form should be used for visits to CHS

Date of visit: _________/_________/__________

(If no is answered for any of these questions, then please include a written explanation of why the answer was "no")

<table>
<tr><th colspan="2" rowspan="2">Questions to be asked during each supervision visit:</th><th colspan="2">Answer</th><th rowspan="2">Why not?</th><th rowspan="2">Recommended actions</th></tr>
<tr><th>Yes</th><th>No</th></tr>
<tr><td colspan="6">Availability of Equipment, Guides, Forms and Tools</td></tr>
<tr><td>4</td><td>Are the following support documents available and up-to-date at this CHS?
<table><tr><td></td><td>In what format are thes documents avaiable?
(ex. Posted on the wall, brochure, fliers, in a file cabinet, on someone's computer…)</td><td>Have you received a new version of these documents from PPMC since the last visit?</td></tr><tr><td>EBS guidelines</td><td></td><td></td></tr><tr><td>List of proposed signals (poster)</td><td></td><td></td></tr></table></td><td></td><td></td><td></td><td></td></tr>
<tr><td rowspan="7">5</td><td>Does the CHS have the necessary equipment available to carry out their EBS reporting duties?</td><td></td><td></td><td rowspan="7"></td><td rowspan="7"></td></tr>
<tr><td>Is there a phone at the CHS?</td><td></td><td></td></tr>
<tr><td>If yes, is it working right now and accessible during out of office hours?</td><td></td><td></td></tr>
<tr><td>Do they have an internet connection?</td><td></td><td></td></tr>
<tr><td>If yes, is the connection available consistently?</td><td></td><td></td></tr>
<tr><td>Is there a computer available for EBS activities?</td><td></td><td></td></tr>
<tr><td>If yes, is it working right now?</td><td></td><td></td></tr>
<tr><td rowspan="2">6</td><td>Does the CHS have all of the necessary forms and tools they need to carry out their EBS reporting duties?</td><td></td><td></td><td rowspan="2"></td><td rowspan="2"></td></tr>
<tr><td>Is there a log book for recording signals?</td><td></td><td></td></tr>
</table>

EBS Supervision Checklist-Commune Health Station (CHS)

This form should be used for visits to CHS

**Name of CHS that is receiving the supportive supervision visit:**	

Date of visit: _________/_________/__________

(If no is answered for any of these questions, then please include a written explanation of why the answer was "no")

	**Questions to be asked during each supervision visit:**	Answer Yes	Answer No	Why not?	Recommended actions
	**Reporting**				
7	**Do Village Health Workers Report signals in person?**				
	If yes, when is somone available from the CHS to receive a report from a VHW? ……………………………………………………………………………………………				
8	**Do Village Helath Workers Report signals by telephone?**				
	If yes, is it by: ☐ landline ☐ mobile phone ☐ Don't Know				
	Does the phone ever stop working? (For example are there off hours?)				
	When is somone available from the CHS to receive a report on the landline or mobile phone from a VHW? ……… …………………………………………………………………				
9	**When Village Health Workers report a signal, what information do they report?** ……… …………………………………………………………………………				
	What information is recorded in the log book when a signal is reported? ……… …………………………………………………………………………				
	Did you provide any feedback to the VHWs on their report?				
	If yes, describe the feedback given: ……… ……………………………………………………………………				
10	**Did you send monthly EBS report to DPMC last month?** *(Please note:* Prior to visiting the province, the DPMC can check to see if the CHS sent their regular EBS reports and review the reports in advance.) *If yes, record the key points and provide feedback to the CHS on the regular report (if necessary):* ……… ……………………………………………………………………				

EBS Supervision Checklist-Commune Health Station (CHS)

This form should be used for visits to CHS

**Name of CHS that is receiving the supportive supervision visit:**	

Date of visit: _________/_________/__________

(If no is answered for any of these questions, then please include a written explanation of why the answer was "no")

	**Questions to be completed based on review of log book:**	Answer		Why not?	Recommended actions
		Yes	No		
11	**Please review the signals in the log book.**<br>**Were any signals/events reported to CHS in the last period/month?**				
	If yes, who reported them?<br>……<br>……………………………………………………………………				
	Is the signal information recorded in the logbook complete for all signals?				
	If no, please describe which information is missing<br>……………………………………………………………………………………………<br>………………………………………………………………………………………………				
	Describe the signals/events (if any):<br>……………………………………………………………………………………………				
	Was feedback provided to Villiage Health Workers regarding their reports?				
	If yes, describe the feedback provided. Was it sufficient?<br>……<br>……………………………………………………………………				
12	**Were any challenges faced in detecting signals/events?**				
	If yes, describe these challenges:<br>……<br>……………………………………………………				
13	**Were any challenges faced in reporting signals/events?**			*If yes, describe these challenges:*	
	No one available to take the call ☐Don't Know				
	Lack of connectivity ☐ Don't Know				
	Didn't know how to report ☐ Don't Know				
	Somebody rejected to take the report ☐Don't Know				
	Other:___________________________				

EBS Supervision Checklist-Commune Health Station (CHS)

Name of CHS that is receiving the supportive supervision visit:	

This form should be used for visits to CHS

Date of visit: _________/_________/__________

(If no is answered for any of these questions, then please include a written explanation of why the answer was "no")

Questions to be asked during each supervision visit:		Answer		Why not?	Recommended actions
		Yes	No		
**Review records/forms**					
14	**Is the logbook for recording signals being used this period?**				
	Is it being used appropriately?				
	What information is recorded in the logbook when a signal is reported?				
15	**Review of the Signal Event Forms for the last month/period:**<br><br>*How many Signal Event Forms were reviewed?...*	*Record the main points and recommendations (if any) regarding completeness and timeliness of the Signal Event Forms after reviewing and discussing with supervisee:*			
16	**Review the EBS Logbook for the last period/month:**<br><br>*How many signals/events were recorded in the logbook for the last period/month?...*	*Record the main points and recommendations (if any) regarding completeness and timeliness of the EBS logbook after reviewing and discuss with supervisee:*			

EBS Supervision Checklist - District Health Center (DHC)

This form should be used for visits to DHC/DPMC

**Name of DHC/DPMC that is receiving the supportive supervision visit:**	

**Province where district is located:**	

Date of visit: _________/_________/__________

(If no is answered for any of these questions, then please include a written explanation of why the answer was "no")

Date of last supportive supervision visit in this district: _____/_____/____

This form is designed for the use of regional/provincial supervisors to conduct supervision visits to the DHC/DPMC level.

**Name of lead supervisor:**		**Position of lead supervisor:**	
**Name of interviewee answering questions during this visit:**		**Position of interviewee:**	

**Questions to be asked during each supervision visit:**		Answer		Why not?	Recommended Actions
		Yes	No		
1	**Has DHC/DPMC designated someone as the focal point person for EBS?**<br>If yes, Name:___________________________________<br>*(Ideally the focal point for EBS should be the person answering these questions)*				
	**Has the staff responsible for EBS changed since the last supportive supervision visit?**<br>If yes, name of new EBS staff:				
	Has new staff received EBS training?				
	**Training:**				
2	**Have at least 2 people in this DHC/DPMC received EBS training?**				
	If EBS staff is being interviewed, ask:<br>*Can you provide the names and contact information (phone, email) of ≥2 PPMC staff to whom signals can be reported?*<br>*Name & Email: ___________________________*<br>*Name & Email: ___________________________*				
	Were any refresher trainings attended by EBS staff from this DHC/DPMC since the last visit?				
3	**Were any training activities related to event based surveillance provided to CHS/ facility staff during the last period/month?**<br>(Including in-class trainings and on-the-job trainings during supervision visits)				
	If yes, which Commune Health Stations, Health Facilities?<br>_______________ ☐ *Trainings* ☐ *Supervision visits* ☐ *Other:_________*				

**Name of DHC/DPMC that is receiving the supportive supervision visit:**	

Date of visit: _________/_________/__________

(If no is answered for any of these questions, then please include a written explanation of why the answer was "no")

	_______________ ☐ *Trainings* ☐ *Supervision visits* ☐ *Other:__________*<br>_______________ ☐ *Trainings* ☐ *Supervision visits* ☐ *Other:__________*<br>_______________ ☐ *Trainings* ☐ *Supervision visits* ☐ *Other:__________*<br>_______________ ☐ *Trainings* ☐ *Supervision visits* ☐ *Other:__________*<br>_______________ ☐ *Trainings* ☐ *Supervision visits* ☐ *Other:__________*				
	Is there a schedule of the dates for refresher training sessions for Commune Health Stations & district facilities/laboratories on EBS?				
	If yes, how frequently is refresher training scheduled?<br>☐ *Monthly* ☐ *Quarterly* ☐ *> Quarterly*				
	Are there plans for educating the community about recognizing and reporting the early warning signals?				

**Name of DHC/DPMC that is receiving the supportive supervision visit:**	

Date of visit: _________/_________/__________

(If no is answered for any of these questions, then please include a written explanation of why the answer was "no")

	**Questions to be asked during each supervision visit:**	Answer Yes	Answer No	Why not?	Recommended actions
	**Availability of Equipment, Guides, Forms and Tools**				
4	**Are the following support documents available and up-to-date at this facility?**				

	In what format are thes documents avaiable? *(ex. Posted on the wall, brochure, fliers, in a file cabinet, on someone's computer…)*	*Have you received a new version of these documents from PPMC since the last visit?*
EBS guidelines		
List of proposed signals (poster)		

		Yes	No	Why not?	Recommended actions
4	**Does the facility have the necessary equipment available to carry out their EBS reporting duties?** *Is there a phone at the DHC/DPMC?*				
	If yes, is it working right now and accessible during out of office hours?				
	Do they have an internet connection?				
	If yes, is the connection available consistently?				
	Is there a computer available for EBS activities?				
	If yes, is it working right now?				
5	**Does the facility have all of the necessary forms and tools they need to carry out their EBS reporting duties?**				
	Is there a form that describes how a signal was verified?				
	Are there forms for monthly summaries of alert signals?				
	Is there a list of the Community Health Stations & Health Facilities/ Laboratories that the DHC/DPMC is responsible for monitoring?				
	Are there forms that can be used for monitoring visits to Community Health Stations or Health Facilities/Laboratories?				
	Is there a way to file forms from monitoring visits for each Community Health Station & Health Facility/Laboratory?				
	Are there Decision Trees for use during risk assessment of an event?				

**Name of DHC/DPMC that is receiving the supportive supervision visit:**	

Date of visit: _________/_________/__________

(If no is answered for any of these questions, then please include a written explanation of why the answer was "no")

	**Questions to be asked during each supervision visit:**	Answer: Yes	Answer: No	Why not?	Recommended actions
	**Reporting**				
6	**During the last period/month, did all CHSs send monthly EBS reports to DHC/DPMC?**				
	If yes, what was the response effort from those reports? ………………………………………………………………………………… ………………………………………………………………………………… *If no, which CHSs did not send monthly reports, and how did you/will you follow up to make sure they send in the forms next month?* ………………………………………………………………………………… ………………………………………………………………………………… *Did you provide any feedback to the CHSs on their report? If yes, describe the feedback given:* ………………………………………………………………………………… …………………………………………………………………………………				
7	**Did you send monthly EBS report to PPMC last month?** *(Please note:* Prior to visiting the province, the PPMC can check to see if the DHC/DPMC sent their regular EBS reports and review the reports in advance.) *If yes, record the key points and provide feedback to the DPMC on the regular report (if necessary):* ………………………………………………………………………………… …………………………………………………………………………………				
8	**Is there a schedule of the dates for monitoring visits to each Community Health Station & Health Facility/Laboratory?**				
	How frequently are monitoring visits scheduled? ☐ Monthly ☐ Quarterly ☐ > Quarterly				
9	**Did DHC/DPMC supervisor(s) make visits to the sites/CHS to supervise/support EBS activities in the last period/month?** *If yes, record the key finding and review the completed checklists and/or trip reports:* ………………………………………………………………………………… ………………………………………………………………………………… *What actions (if any) were taken?* ………………………………………………………………………………… …………………………………………………………………………………				

EBS Supervision Checklist - District Health Center (DHC)

This form should be used for visits to DHC/DPMC

**Name of DHC/DPMC that is receiving the supportive supervision visit:**	

Date of visit: _________/_________/__________

(If no is answered for any of these questions, then please include a written explanation of why the answer was "no")

**Questions to be asked during each supervision visit:**		Answer		Why not?	Recommended actions
		Yes	No		
10	**Please review the signals in the log book.**<br>**Were any signals/events reported to DHC/DPMC in the last period/month?**				
	If yes, who reported them?<br>…………………………………………………………………………………………<br>…………………………………………………………………………………………				
	Is the signal information recorded in the logbook complete for all signals?				
	If no, please describe which information is missing<br>…………………………………………………………………………………………<br>…………………………………………………………………………………………<br>*Describe the signals/events (if any):*<br>…………………………………………………………………………………………<br>*What measures were taken to respond to the reported signals/events?*<br>…………………………………………………………………………………………<br>…………………………………………………………………………………………				
11	**Were any challenges faced in detecting signals/events?**<br>*If yes, describe these challenges:*<br>…………………………………………………………………………………………<br>………………………………………………………………………………				
12	**Were any challenges faced in reporting signals/events?**<br>No one available to take the call ☐Don't Know			*If yes, describe these challenges:*	
	Lack of onnectivity ☐ Don't Know				
	Didn't know how to report ☐ Don't Know				
	Somebody rejected to take the report ☐Don't Know				
13	**Were any challenges faced in responding to signals/events?**<br>*If yes, describe these challenges:*<br>…………………………………………………………………………………………<br>………………………………………………………………………				

**Name of DHC/DPMC that is receiving the supportive supervision visit:**	

Date of visit: _________/_________/__________

(If no is answered for any of these questions, then please include a written explanation of why the answer was "no")

	**Questions to be asked during each supervision visit:**	Answer		Why not?	Recommended actions
		Yes	No		
**Review records/forms**					
14	**Is the logbook for recording signals being used this period?**				
	Is it being used appropriately?				
	What information is recorded in the logbook when a signal is reported?				
15	**Review of the Signal Event Forms for the last month/period:** *How many Signal Event Forms were reviewed?...*	*Record the main points and recommendations (if any) regarding completeness and timeliness of the Signal Event Forms after reviewing and discussing with supervisee:*			
16	**Review the EBS Logbook for the last period/month:** *How many signals/events were recorded in the logbook for the last period/month?..*	*Record the main points and recommendations (if any) regarding completeness and timeliness of the EBS logbook after reviewing and discuss with supervisee:*			

EBS Supervision Checklist-Provincial (PPMC)

**Name of PPMC that is receiving the supportive supervision visit:**	

**Region where PPMC located:**	

This form should be used for visits to PPMC

Date of visit: _________/_________/__________

(If no is answered for any of these questions, then please include a written explanation of why the answer was "no")

Date of last supportive supervision visit in this province: _____/_____/____

This form is designed for the use of regional/provincial supervisors to conduct supervision visits to the PPMC level.

**Name of lead supervisor:**		**Position of lead supervisor:**	
**Name of interviewee answering questions during this visit:**		**Position of interviewee:**	

Questions to be asked during each supervision visit:		Answer		Why not?	Recommended Actions
		Yes	No		
1	**Has PPMC designated someone as the focal point person for EBS?**<br>If yes, Name:___________________________________<br>*(Ideally the focal point for EBS should be the person answering these questions)*				
	**Has the staff responsible for EBS changed since the last supportive supervision visit?**<br>If yes, name of new EBS staff:				
	Has new staff received EBS training?				
	**Training:**				
2	**Have at least 2 people in this PPMC received EBS training?**				
	If EBS staff is being interviewed, ask:<br>*Can you provide the names and contact information (phone, email) of >2 Regional Institute staff to whom signals can be reported?*<br>*Name & Email:* ___________________________<br>*Name & Email:* ___________________________				
	Were any refresher trainings attended by EBS staff from this PPMC since the last visit?				
3	**Were any training activities related to event based surveillance provided to District level staff in this Province during the last period/month?**<br>(Including in-class trainings and on-the-job trainings during supervision visits)				
	If yes, list the DPMCs/DHCs and how they received training?<br>_______________ ☐ *Trainings* ☐ *Supervision visits* ☐ *Other:*_________				

**Name of PPMC that is receiving the supportive supervision visit:**	

Date of visit: _________/_________/__________

(If no is answered for any of these questions, then please include a written explanation of why the answer was "no")

	_______________ ☐ *Trainings* ☐ *Supervision visits* ☐ *Other:_________*<br>_______________ ☐ *Trainings* ☐ *Supervision visits* ☐ *Other:_________*<br>_______________ ☐ *Trainings* ☐ *Supervision visits* ☐ *Other:_________*<br>_______________ ☐ *Trainings* ☐ *Supervision visits* ☐ *Other:_________*<br>_______________ ☐ *Trainings* ☐ *Supervision visits* ☐ *Other:_________*			
	Is there a schedule of the dates for refresher training sessions for DHCs/DPMCs on EBS?			
	If yes, how frequently is refresher training scheduled?<br>☐ *Monthly* ☐ *Quarterly* ☐ *> Quarterly*			

EBS Supervision Checklist-Provincial (PPMC)

This form should be used for visits to PPMC

**Name of PPMC that is receiving the supportive supervision visit:**	

Date of visit: _________/_________/__________

(If no is answered for any of these questions, then please include a written explanation of why the answer was "no")

	**Questions to be asked during each supervision visit:**	Answer Yes	Answer No	Why not?	Recommended actions
	**Availability of Equipment, Guides, Forms and Tools**				
4	**Are the following support documents available and up-to-date at this PPMC?**				
	(sub-table below)				
4	**Does the PPMC have the necessary equipment available to carry out their EBS reporting duties?**				
	Is there a log book for recording signals?				
	If yes, is it being used?				
	Is it electronic or paper based?				
	Is there a phone at the PPMC?				
	If yes, is it working right now and accessible during out of office hours?				
	Do they have an internet connection?				
	If yes, is the connection available consistently?				
	Is there a computer available for EBS activities?				
	If yes, is it working right now?				
5	**Does the PPMC have all of the necessary forms and tools they need to carry out their EBS reporting duties?**				
	Is there a form that describes how a signal was verified?				
	Are there forms for monthly summaries of alert signals?				
	Is there a list of the DHC/DPMCs that the PPMC is responsible for monitoring?				
	Are there forms that can be used for monitoring visits to DHCs/DPMCs?				
	Is there a way to file forms from monitoring visits for each DHCs/DPMCs?				
	Does the PPMC have a Decision Trees for use during risk the assessment of an event?				

Sub-table for question 4 (support documents):

	In what format are thes documents avaiable? *(ex. Posted on the wall, brochure, fliers, in a file cabinet, on someone's computer…)*	*Have you received a new version of these documents from GDPM since the last visit?*
EBS guidelines		
List of proposed signals (poster)		

EBS Supervision Checklist-Provincial (PPMC)

This form should be used for visits to PPMC

**Name of PPMC that is receiving the supportive supervision visit:**	

Date of visit: _________/_________/__________

(If no is answered for any of these questions, then please include a written explanation of why the answer was "no")

	**Questions to be asked during each supervision visit:**	Answer Yes	Answer No	Why not?	Recommended actions
	**Reporting**				
6	**During the last period/month, did all DHC/DPMC send monthly EBS reports to PPMC?**				
	If yes, what was the response effort from those reports? ………………………………………………………………………… ………………………………………………………………………… *If no, which DHC/DPMCs did not send monthly reports, and how did you/will you follow up to make sure they send in the forms next month?* ………………………………………………………………………… ………………………………………………………………………… *Did you provide any feedback to the DHC/DPMCs on their report? If yes, describe the feedback given:* ………………………………………………………………………… …………………………………………………………………………				
7	**Did you send monthly EBS report to the RI last month?** *(Please note:* Prior to visiting the province, the RI can check to see if the PPMC sent their regular EBS reports and review the reports in advance.) *If yes, record the key points and provide feedback to the PPMC on the regular report (if necessary):* ………………………………………………………………………… …………………………………………………………………………				
8	**Is there a schedule of the dates for monitoring visits to each DHC & DPMC?**				
	How frequently are monitoring visits scheduled? ☐ Monthly ☐ Quarterly ☐ > Quarterly				
9	**Did PPMC supervisor(s) make visits to the DHCs/DPMCs to supervise/support EBS activities in the last period/month?** *If yes, record the key finding and review the completed checklists and/or trip reports:* ………………………………………………………………………… ………………………………………………………………………… *What actions (if any) were taken?* ………………………………………………………………………… …………………………………………………………………………				

EBS Supervision Checklist-Provincial (PPMC)

This form should be used for visits to PPMC

**Name of PPMC that is receiving the supportive supervision visit:**	

Date of visit: _________/_________/__________

(If no is answered for any of these questions, then please include a written explanation of why the answer was "no")

	**Questions to be asked during each supervision visit:**	Answer		Why not?	Recommended actions
		Yes	No		
10	**Please review the signals in the log book.**<br>**Were any signals/events reported to PPMC in the last period/month?**				
	If yes, who reported them?<br>…………………………………………………………………………………<br>…………………………………………………………………………………				
	Is the signal information recorded in the logbook complete for all signals?				
	If no, please describe which information is missing<br>…………………………………………………………………………………<br>…………………………………………………………………………………<br>*Describe the signals/events (if any):*<br>…………………………………………………………………………………<br>*What measures were taken to respond to the reported signals/events?*<br>…………………………………………………………………………………<br>…………………………………………………………………………………				
11	**Were any challenges faced in detecting signals/events?**<br>*If yes, describe these challenges:*<br>…………………………………………………………………………………<br>……………………………………………………………………				
12	**Were any challenges faced in reporting signals/events?**			*If yes, describe these challenges:*	
	No one available to take the call ☐Don't Know				
	Lack of onnectivity ☐ Don't Know				
	Didn't know how to report ☐ Don't Know				
	Somebody rejected to take the report ☐Don't Know				
	Other:____				
13	**Were any challenges faced in responding to signals/events?**<br>*If yes, describe these challenges:*<br>…………………………………………………………………………………<br>……………………………………………………………………				

EBS Supervision Checklist-Provincial (PPMC)

Name of PPMC that is receiving the supportive supervision visit:	

This form should be used for visits to PPMC

Date of visit: _________/_________/__________

(If no is answered for any of these questions, then please include a written explanation of why the answer was "no")

**Questions to be asked during each supervision visit:**		Answer		Why not?	Recommended actions
		Yes	No		
**Review records/forms**					
14	**Is the logbook for recording signals being used this period?**				
	Is it being used appropriately?				
	What information is recorded in the logbook when a signal is reported?				
15	**Review of the Signal Event Forms for the last month/period:**<br><br>*How many Signal Event Forms were reviewed?...*	*Record the main points and recommendations (if any) regarding completeness and timeliness of the Signal Event Forms after reviewing and discussing with supervisee:*			
16	**Review the EBS Logbook for the last period/month:**<br><br>*How many signals/events were recorded in the logbook for the last period/month?...*	*Record the main points and recommendations (if any) regarding completeness and timeliness of the EBS logbook after reviewing and discuss with supervisee:*			

DESK REVIEW TOOL

***Instruction**: Assessment team should be responsible to collect the following information in advance or during the field trip at each RI, PPMC, DPMC and CHS sampled.*

Part I: General Information

Name of RI/PPMC/DPMC/CHS assessed	
Date of completing form *(dd/mm/yyyy)*	
Name/Title of staff(s) provided information	
Name/Title of staff(s) completed this form	

Part II: Indicators related to implementing the EBS such as training, system coverage, supervision visits

***Instruction**: In order to collect information for the Part I, assessment team should collect EBS plans and progress reports as well as interview EBS focal points at different levels.*

2.1. Training

Indicator	Plan	Progress up-to-date	# of participants	Comments
Only at GDPM and RIs				
1. # of TOT trainings				
Only at PPMC				
2. # of trainings by provinces				
Only at DPMC				
3. # of trainings by districts				
Only at CHS				
4. # of trainings by communes				
At all levels				
5. # of village health workers/collaborators trained on EBS			NA	
6. # of hospitals trained on EBS			NA	
7. # of pharmacies trained on EBS			NA	
8. # of schools trained on EBS			NA	
9. # of industrial zones trained on EBS			NA	

2.2. System coverage

Indicator	Plan	Progress up-to-date	Comments
System Coverage			
1. # of districts implementing EBS			
2. # of communes implementing EBS			
Supervision Visit			
1. # of supportive supervision visits conducted			

Part III. Indicators collected from the monthly summary report (Annex 9)

3.1. Monthly Summary Report

***Instruction**: Collect monthly summary reports and then validate the number reported with the original data sources such as logbooks, event verification forms, etc. Specifically,*

- *At provincial level: review the original reports from districts, recalculate the indicators & compare them with the ones reported to RIs and GDPM*
- *At district level: review original reports from communes and district's logbook, recalculate the indicators & compare them with the ones reported to PPMC*
- *At commune level: review commune's logbook, recalculate the indicators & compare them with the ones reported to DPMC.*

Indicator	Reported Value	Verified Value	Comments
1. Total number of alert signals reported			
2. Number of alert signals triaged			
3. Number of alert signals reported to upper level after triaged			
4. Number of triaged signals verified			
5. Number of alert signals become events			
6. Number of events assessed risk			
7. Number of events responded			

3.2. Checklist for Review of Logbooks

Component to review	Yes/No	Comments
1. If a signal is recorded, all the necessary information is written in the appropriate column for each row	☐ Yes ☐ No	
2. The handwriting is clear and legible	☐ Yes ☐ No	
3. Dates entered for each row are logical	☐ Yes ☐ No	
4. If verification result is filled, is triage results coded as "3"- reported *signal is in the list of priority alert signals, is verified and reported.*	☐ Yes ☐ No	
Only at PPMC		
5. If risk assessment result is filled, is verification results coded as "2"- *Become an event*	☐ Yes ☐ No	

Guide for Focus Group Discussion with VHWs & DPMC focal points

Focus Group Facilitator's Name	FG #

Focus group information			
Place of interview:			Date of focus group
Province	District	Commune	

Participant information						
#	Sex	Age	Education level	Participant's occupation	Participant's Role in EBS *(and number of months involved in EBS)*	Consent *(to record))*
1	☐ Male<br>☐ Female	☐ less than 20 yr<br>☐ 20-39 yr<br>☐ 40-59 yr<br>☐ 60 yr or older	☐ Primary school<br>☐ Secondary school<br>☐ High school/equivalent<br>☐ College and more			☐ Yes<br>☐ No
2	☐ Male<br>☐ Female	☐ less than 20 yr<br>☐ 20-39 yr<br>☐ 40-59 yr<br>☐ 60 yr or older	☐ Primary school<br>☐ Secondary school<br>☐ High school/equivalent<br>☐ College and more			☐ Yes<br>☐ No
3	☐ Male<br>☐ Female	☐ less than 20 yr<br>☐ 20-39 yr<br>☐ 40-59 yr<br>☐ 60 yr or older	☐ Primary school<br>☐ Secondary school<br>☐ High school/equivalent<br>☐ College and more			☐ Yes<br>☐ No
4	☐ Male<br>☐ Female	☐ less than 20 yr<br>☐ 20-39 yr<br>☐ 40-59 yr<br>☐ 60 yr or older	☐ Primary school<br>☐ Secondary school<br>☐ High school/equivalent<br>☐ College and more			☐ Yes<br>☐ No
5	☐ Male<br>☐ Female	☐ less than 20 yr<br>☐ 20-39 yr<br>☐ 40-59 yr<br>☐ 60 yr or older	☐ Primary school<br>☐ Secondary school<br>☐ High school/equivalent<br>☐ College and more			☐ Yes<br>☐ No
6	☐ Male<br>☐ Female	☐ less than 20 yr<br>☐ 20-39 yr<br>☐ 40-59 yr<br>☐ 60 yr or older	☐ Primary school<br>☐ Secondary school<br>☐ High school/equivalent<br>☐ College and more			☐ Yes<br>☐ No
7	☐ Male<br>☐ Female	☐ less than 20 yr<br>☐ 20-39 yr<br>☐ 40-59 yr<br>☐ 60 yr or older	☐ Primary school<br>☐ Secondary school<br>☐ High school/equivalent<br>☐ College and more			☐ Yes<br>☐ No
8	☐ Male<br>☐ Female	☐ less than 20 yr<br>☐ 20-39 yr<br>☐ 40-59 yr<br>☐ 60 yr or older	☐ Primary school<br>☐ Secondary school<br>☐ High school/equivalent<br>☐ College and more			☐ Yes<br>☐ No
9	☐ Male<br>☐ Female	☐ less than 20 yr<br>☐ 20-39 yr<br>☐ 40-59 yr<br>☐ 60 yr or older	☐ Primary school<br>☐ Secondary school<br>☐ High school/equivalent<br>☐ College and more			☐ Yes<br>☐ No
10	☐ Male<br>☐ Female	☐ less than 20 yr<br>☐ 20-39 yr<br>☐ 40-59 yr<br>☐ 60 yr or older	☐ Primary school<br>☐ Secondary school<br>☐ High school/equivalent<br>☐ College and more			☐ Yes<br>☐ No

Guide for Focus Group Discussion with VHWs & DPMC focal points

Focus Group Facilitator's Name	FG #

Introduction

estimated time for Focus Group Duration: 1- 1.5 Hours

Thank you for participating in this focus group for the Event Base Surveillance pilot in Vietnam. We would like to ask you about your opinions about the EBS system based on your experience. This focus group is being done as part of a larger effort to document the lessons we can learn from the pilot of the EBS in four provinces in Vietnam. Your answers will be used to help us come up with recommendations for the government to improve the EBS processes, trainings and reporting tools, and document the benefits and challenges of EBS before it is rolled out nationally across Vietnam. Your responses will also help CDC understand the benefits of EBS in Vietnam, which may help other countries who are considering implementing EBS.

Consent to record the conversation

This is not considered a research study, however, if you agree, **we would like to record this conversation** so we can accurately summarize your responses with the responses we get from other groups in all four provinces over the next two weeks. If at any time you are not comfortable answering a question, you are welcome to skip the question. If at any time you would like to leave the focus group, you may do so.

- Is it okay with you if we record today's conversation to help us with note taking and summarizing what you say with what others say about the EBS?
  - *[Wait for confirmation from all. Did they agree? Check <u>YES</u> or <u>No</u> in the sign in sheets]*
- Do you understand that you may skip any questions you would like and that you can end the interview at any time?
  - *[Wait for their confirmation from all. Did they agree? Check <u>YES</u> or <u>No</u> in the sign in sheets]*

Questions

Fidelity to implementation

1. In one or two sentences, tell us about how you have been involved in implementing EBS so far?

<u>Ask this question if you are interviewing VHWs. If not, skip and move to Question 3</u>

2. What challenges did you or other volunteers you know come across when it came to detecting and reporting signals for EBS?
   PROBE: Do you feel confident in your ability to identify signals? Do you know how to report signals? Do you feel confident that informants will approach you to report health related events in your community?

Focus Group Facilitator's Name	FG #

<u>Ask this question if you are interviewing district representatives. If not, skip and move to Question 4</u>

3. What challenges did you come across when it came to verifying and reporting events?
PROBE: Do you feel confident in your ability to verify events? Do you know how to report events?

Timeliness

4. In your opinion, do you think the EBS has helped for early detection of events and outbreaks?
Probe: For example, do you think it has helped to reduce the time between the time an event or outbreak happens and the time the health authorities are notified?

Costs

5. Approximately how much time do you believe you spend each week working on the EBS?
Probe: some weeks it may be more, some weeks it may be less, what is the range?

Perceived value of EBS / Acceptability / willingness to participate

6. In your opinion is it worth the efforts to implement EBS?
Probe: Why?

---

7. What do you think are the biggest strengths of the EBS?

---

8. How willing are you to put support the EBS for a few hours every week in the future?

<u>Ask this question if you are interviewing VHWs. If not, skip and move to Question 10</u>

9. What motivates you as a commune health volunteer?
PROBE: Why did you choose to become a community health volunteer?

Guide for Focus Group Discussion with VHWs & DPMC focal points

Focus Group Facilitator's Name	FG #

Reporting

Ask this question if you are interviewing District's participants. If not, skip and move to Question 12

10. When you identify an event, tell us how do you register it into the eCDS?
    PROBE: Let's take an example: You have a measles case from a school by the community, How will this be reported into the public health system? Another example: You have a measles case at a hospital. How will this be reported into the public health system? Do you fill an eCDS's case report form? Do you fill an eCDS outbreak form?

Ask this question if you are interviewing district's participants.

11. Do you think eCDS and EBS are overlapping? If Yes, tell us How?

Lessons for future roll out

12. Based on your experience with the EBS so far, what do you think is important for us to think about to make sure EBS can continue in the future?

---

13. What do you think are the most important limitations of the EBS?

Ask this question if you are interviewing VHWs.

14. How do you can increase your pool of informants in the community?
    PROBE: Why did you choose to become a community health volunteer?

Is there anything you would like to tell us about the EBS system as you have experienced it so far that you have not had a chance to say yet?

Guide for Focus Group Discussion with Information Sources in the Community

Focus Group Facilitator's Name	FG #

Focus group information			
Place of interview:			Date of focus group
Province	District	Commune	

Participant information						
#	Sex	Age	Education level	Participant's occupation	Type of source	Consent *(to record))*
1	☐ Male<br>☐ Female	☐ less than 20 yr<br>☐ 20-39 yr<br>☐ 40-59 yr<br>☐ 60 yr or older	☐ Primary school<br>☐ Secondary school<br>☐ High school/equivalent<br>☐ College and more		☐ School teacher<br>☐ Pharmacy<br>☐ Private clinic<br>☐ Workplace<br>☐ Other ____________	☐ Yes<br>☐ No
2	☐ Male<br>☐ Female	☐ less than 20 yr<br>☐ 20-39 yr<br>☐ 40-59 yr<br>☐ 60 yr or older	☐ Primary school<br>☐ Secondary school<br>☐ High school/equivalent<br>☐ College and more		☐ School teacher<br>☐ Pharmacy<br>☐ Private clinic<br>☐ Workplace<br>☐ Other ____________	☐ Yes<br>☐ No
3	☐ Male<br>☐ Female	☐ less than 20 yr<br>☐ 20-39 yr<br>☐ 40-59 yr<br>☐ 60 yr or older	☐ Primary school<br>☐ Secondary school<br>☐ High school/equivalent<br>☐ College and more		☐ School teacher<br>☐ Pharmacy<br>☐ Private clinic<br>☐ Workplace<br>☐ Other ____________	☐ Yes<br>☐ No
4	☐ Male<br>☐ Female	☐ less than 20 yr<br>☐ 20-39 yr<br>☐ 40-59 yr<br>☐ 60 yr or older	☐ Primary school<br>☐ Secondary school<br>☐ High school/equivalent<br>☐ College and more		☐ School teacher<br>☐ Pharmacy<br>☐ Private clinic<br>☐ Workplace<br>☐ Other ____________	☐ Yes<br>☐ No
5	☐ Male<br>☐ Female	☐ less than 20 yr<br>☐ 20-39 yr<br>☐ 40-59 yr<br>☐ 60 yr or older	☐ Primary school<br>☐ Secondary school<br>☐ High school/equivalent<br>☐ College and more		☐ School teacher<br>☐ Pharmacy<br>☐ Private clinic<br>☐ Workplace<br>☐ Other ____________	☐ Yes<br>☐ No
6	☐ Male<br>☐ Female	☐ less than 20 yr<br>☐ 20-39 yr<br>☐ 40-59 yr<br>☐ 60 yr or older	☐ Primary school<br>☐ Secondary school<br>☐ High school/equivalent<br>☐ College and more		☐ School teacher<br>☐ Pharmacy<br>☐ Private clinic<br>☐ Workplace<br>☐ Other ____________	☐ Yes<br>☐ No
7	☐ Male<br>☐ Female	☐ less than 20 yr<br>☐ 20-39 yr<br>☐ 40-59 yr<br>☐ 60 yr or older	☐ Primary school<br>☐ Secondary school<br>☐ High school/equivalent<br>☐ College and more		☐ School teacher<br>☐ Pharmacy<br>☐ Private clinic<br>☐ Workplace<br>☐ Other ____________	☐ Yes<br>☐ No

Guide for Focus Group Discussion with Information Sources in the Community

Focus Group Facilitator's Name	FG #

Introduction estimated time for Focus Group Duration: 1- 1.5 Hours

Thank you for participating in this focus group for the Event Base Surveillance pilot in Vietnam. We would like to ask you about your opinions about the EBS system based on your experience. This focus group is being done as part of a larger effort to document the lessons we can learn from the pilot of the EBS in four provinces in Vietnam. Your answers will be used to help us come up with recommendations for the government to improve the EBS processes, trainings and reporting tools, and document the benefits and challenges of EBS before it is rolled out nationally across Vietnam. Your responses will also help CDC understand the benefits of EBS in Vietnam, which may help other countries who are considering implementing EBS.

Consent to record the conversation

This is not considered a research study, however, if you agree, **we would like to record this conversation** so we can accurately summarize your responses with the responses we get from other groups in all four provinces over the next two weeks. If at any time you are not comfortable answering a question, you are welcome to skip the question. If at any time you would like to leave the focus group, you may do so.

- Is it okay with you if we record today's conversation to help us with note taking and summarizing what you say with what others say about the EBS?
  - *[Wait for confirmation from all. Did they agree? Check YES or No in the sign in sheets]*
- Do you understand that you may skip any questions you would like and that you can end the interview at any time?
  - *[Wait for their confirmation from all. Did they agree? Check YES or No in the sign in sheets]*

Questions

Current Knowledge of EBS

1. Please tell us How much do you know about "Event-Based Surveillance (EBS)"
   NOTE: Some people may not recognize the term "EBS" even when they know or are aware of this surveillance
   PROBE: Do you know that you can report some health related event to Commune Health Station or commune health volunteer?

---

2. Could you tell us How and when did you know that is possible to report some health related events ("signals") to the Commune Health Station or Village Health volunteers?
   (This question should be asked only to people who said they know about this surveillance)
   NOTE: Some people may not recognize the term "EBS" even when they know or are aware of this surveillance
   PROBE: Communicated by the VHWs, read the posters, training, etc.) Have you received any training in EBS before? If YES, who trained you?

3. Could you tell us what health related events ("signals") you should be looking for reporting to Commune Health Station or Village Health volunteers?
   (This question should be asked only to people who said they know about EBS)

Reporting signals

4. Have you ever reported any health related event ("signal"') to your village health workers or commune health stations? If Yes, what did you report?
   PROBE: What kind of signal(s) did you report? To whom did you report the signal(s)? Why did you report the signal(s)? Did you have any problem to report the signal(s)?

---

5. Tell us which of the following signals you have seen or known in the last three (3) months in your place [school, pharmacy, workplace, community] even if you did not report them
   (Read all signals for EBS in the community).
   PROBE: If you have found any of these health related events ["signals"] tell us How did you find them?

Guide for Focus Group Discussion with Information Sources in the Community

Focus Group Facilitator's Name	FG #

Perceived value of EBS / Acceptability / willingness to participate

6. Do you think it's beneficial to implement EBS in your commune? If yes, please list some benefit of EBS.

---

7. If you detect a signal in the future, are you willing to report to VHWs or CHSs?

---

8. What encourage you to report these health related events ('signals")?

---

9. Are there additional people in your community that can serve as an informant for these health related events?

Is there anything you would like to tell us about the EBS system as you have experienced it so far that you have not had a chance to say yet?

Key Informant Interview Guide for the EBS Evaluation

to be filled in by the interviewer (or note taker) during the Key Informant Interviews

Interviewer's Name	Date	Interview #

Place of interview:			Sex of interviewee (male or female)	Interviewee's position	Number of Months involved with EBS	Consent to record the interview
Province name	District name	Commune name				
						☐ Yes<br>☐ No

Introduction:

Thank you for participating in this key informant interview for the Event Base Surveillance pilot in Vietnam. We would like to ask you about your opinions about the EBS system based on your experience. This interview is being done as part of a larger effort to document the lessons we can learn from the pilot of the EBS in four provinces in Vietnam. As part of this evaluation we are doing Key Informant interviews with EBS Focal Points at Commune Health Stations, District PMC, Province PMC, Regional Institutes, and with GDPM. Your answers will be used to help us come up with recommendations for the government to improve the EBS processes, trainings and reporting tools, and document the benefits and challenges of EBS before it is rolled out nationally across Vietnam. Your responses will also help CDC understand the benefits of EBS in Vietnam, which may help other countries who are considering implementing EBS.

Consent to record the conversation:

This is not considered a research study, however, if you agree, **we would like to record this conversation** so we can accurately summarize your responses with the responses we get from other people we interview in all four provinces over the next two weeks. If at any time you are not comfortable answering a question, you are welcome to skip the question. If at any time you would like to end the interview you may do so.

- Is it okay with you if we record today's conversation to help us with note taking and summarizing what you say with what others say about the EBS?
  - *[Did he/she agree? Check YES or No]*
- Do you understand that you may skip any questions you would like and that you can end the interview at any time?
  - *[Did he/she agree? Check YES or No]*

**Interviewer's Name**	**Date**	**Interview #**

---

1. **Fidelity to implementation** – *These first few questions are about how well the EBS is being implemented based on what you have seen so far (since September 2016).*
   a. In your opinion and based on your experience in your [province/district/commune], How well do you think EBS has been implemented according to the guidelines?

   b. Can you briefly tell us about your experience tasks in the EBS process since it was started?

      *PROBE: At **Province Level:** How do you conduct the risk assessment for events that are verified? How do you decide when the PROVINCE level needs to do risk assessment vs. when the DISTRICT does a risk assessment? How do you determine what kind of response is needed and if a response team needs to be deployed?*

      *PROBE: At **District Level**: What is your process for doing verification? How do you decide if a signal needs to be verified?*

      *PROBE: At **Commune Health Station**: How do you conduct the triage of signals? How do you report a signal to the district?*

   c. What challenges do you believe might have prevented perfect adherence to EBS protocol in the past 6 months?

      PROBE: This may include any challenges from detecting a signal, reporting, triage, verification, Risk assessment to Response. Interviewer should know (use cheat-sheet if necessary) what roles the interviewee is responsible for.

      Another way to ask: What do you think are the most important limitations or barriers to successful implementation of the EBS?

   d. What challenges did you or other people you work with to implement EBS have in using the communication materials, log books or preparing summary reports?

---

2. **Timeliness:** *These next two questions ask you to think about before EBS and now.*
   a. In your opinion, do you think the EBS has helped for early detection of events and outbreaks?

      PROBE: For example, do you think it has helped to reduce the time between the time an event or outbreak happens and the time the health authorities are notified? Please tell us briefly how it was before and how it is now.

   a. In your opinion, do you think the EBS has been able to reduce the time until a response is implemented to face a potential health threat? Please tell us briefly how it was before and how it is now.

3. **Perceived value of EBS / Acceptability / willingness to participate:** *These questions ask about your experience.*
   a. In your opinion, what is (or are) the most important benefits (or strengths) of the EBS system?

   b. In your opinion is the effort it takes to implement EBS in your area worth the potential benefits that can come from EBS?

   c. In your experience do most of the people you know who are working on EBS think it is a worth the costs and time they spend on it?

---

4. **Costs:** *The next few questions ask about perceived costs of the EBS.*
   a. How much time do you believe you spend each week working on the EBS?

   b. What do you think are some of the important costs of implementing the EBS in your area [province/district/commune] that need to be budgeted for if we want to make EBS sustainable in the long term?

---

5. **Lessons for future roll out:** *These questions will help us provide recommendations for future roll out.*
   a. Thinking about each of these following items, what recommendations do you have to help improve any of the following?

	If the interviewee says they had no exposure, put a check mark here:	What changes do you recommend?	Other comments?
The technical guidelines			
Training			
Log Book			
Monthly Summary Reports			
Communications materials			

   b. What do you think were the most important facilitators to make sure the EBS is working in your area?

   c. Based on your experience with the EBS so far, what do you think is important for us to think about to make sure EBS can continue in the future?

Interviewer's Name	Date	Interview #

d. Are you or your colleagues at this level responsible for "response in the EBS?
   - IF YES ASK: How do you determine when an event requires response?
     - Probe: What factors do you take into consideration to decide if you should respond and what kind of response is needed (ex. Lab confirmation)
     - Probe: How do you respond? What does response at this level look like?

Interviewer's Name	Date	Interview #

Key Informant Interview Guide for the EBS Evaluation

to be filled in by the interviewer (or note taker) during the Key Informant Interviews

---

Key Informant Interview Guide for EBS in hospital

Place of interview:			Interviewee's position	Sex of interviewee (male or female)	Number of Months involved with EBS
Province name	District name	Hospital name			

Introduction:

Thank you for participating in this key informant interview for the Event Base Surveillance pilot in Vietnam. We would like to ask you about your opinions about the EBS system based on your experience. This interview is being done as part of a larger effort to document the lessons we can learn from the pilot of the EBS in four provinces in Vietnam. As part of this evaluation we are doing Key Informant interviews with EBS Focal Points at Commune Health Stations, District PMC, Province PMC, Regional Institutes, and with GDPM. Your answers will be used to help us come up with recommendations for the government to improve the EBS processes, trainings and reporting tools, and document the benefits and challenges of EBS before it is rolled out nationally across Vietnam. Your responses will also help CDC understand the benefits of EBS in Vietnam, which may help other countries who are considering implementing EBS.

---

1. **Fidelity to implementation** – *These first few questions are about how well the EBS is being implemented based on what you have seen so far (since September 2016).*
    a. In your opinion and based on your experience in your hospital, How well do you think EBS has been implemented according to the guidelines?

    b. Can you briefly tell us about your experience in detecting signals for EBS since it was started?

    c. What challenges do you believe might have prevented perfect adherence to EBS protocol (focused in detection of signals) in the past 6 months in your hospital?

        PROBE: Did you have any problems to understand signals definition for hospitals? (Ask for each of the three signals)

Interviewer's Name	Date	Interview #

Key Informant Interview Guide for the EBS Evaluation

to be filled in by the interviewer (or note taker) during the Key Informant Interviews

---

2. **Perceived value of EBS / Acceptability / willingness to participate:** *These questions ask about your experience.*
   a. In your opinion, what is (or are) the most important benefits (or strengths) of the EBS system?

   b. In your opinion is the effort it takes to implement EBS in your hospital worth the potential benefits that can come from EBS?

---

3. **Lessons for future roll out:** *These questions will help us provide recommendations for future roll out.*
   a. Thinking about each of these following items, what recommendations do you have to help improve any of the following?

	If the interviewee says they had no exposure, put a check mark here:	What changes do you recommend?	Other comments?
The technical guidelines			
Training			
Communications materials			

   b. Based on your experience with the EBS so far, what do you think is important for us to think about to make sure EBS can continue in the future?

TIMELINESS FORM

Instructions: All districts in the pilot provinces will receive the timeliness table. Districts will:

- Examine their logbook and list **the events (<u>not</u> signals)**
- Only for the listed events further data will be entered

#	Districts examine their own logbook				Districts call the organizations which firstly reported/informed signals of the event to your district to check their logbook	
	**Content of events**<br><br>**(2)**	**Date & Time when event was registered at the district level**<br>(time/day/month/year)<br>**(3)**	**Date & Time when a response for the event was initiated**<br>(time/day/month/year)<br>**(4)**	**Response activities implemented**<br>(see below)<br>**(5)**	**Date & Time when signal was registered in the CHS logbook**<br>(time/day/month/year)<br>**(6)**	**Date & Time of onset of signal/symptoms (signal appeared)**<br>(time/day/month/year)<br>**(7)**
**(1)**						

Steps to fill out each column:

1. **Districts have to examine their own logbook**

a) Column (1): Write consecutive numbers in each district

b) Column (2): Copy the original information related to the content of the event in their logbook and paste here

c) Column (3): Register the date and time when the event was registered at the district level (check the district logbook) [time/day/month/year].

d) Column (4): Register the date and time when a response for the event was initiated (check the district logbook) [time/day/month/year]

e) Column (5): Write the most important response activities implemented, e.g. “vaccination”, “health education”, “outbreak investigation”, “laboratory testing”, “containment”, “risk communication”, etc.

2. **Districts call the organizations which firstly reported/informed signals of the event to your district to check their logbook**

a) Column (6): Register the date and time when the signal of this event was registered in the CHS logbook [time/day/month/year]

b) Column (7): Register the date and time of onset of signal and symptoms (when the signal of this event appeared) [time/day/month/year]. This information is usually extracted from the column #4 in the logbook

TIMELINESS FORM

General Information

Name of unit provided information	
Province/District/Commune	
Date of completing form *(dd/mm/yyyy)*	
Name/Title of staff(s) provided information	

This data collection tool is designed to collect information to assess the timeliness of EBS. Please help to fill out the missing information just ***for events*** ***(NOT for signals)*** *that have occurred in your district in the below table.*

# (1)	Districts examine their own logbook				Districts call the organizations which firstly reported/informed signals of the event to your district to check their logbook	
	**Content of events** (2)	**Date & Time when event was registered at the district level** (time/day/month/year) **(3)**	**Date & Time when a response for the event was initiated** (time/day/month/year) **(4)**	**Response activities implemented** (see below) **(5)**	**Date & Time when signal was registered in the CHS logbook** (time/day/month/year) **(6)**	**Date & Time of onset of signal/symptoms (signal appeared)** (time/day/month/year) **(7)**

SURVEY OF EBS ACCEPTABILITY. A SELF-ADMINISTERED QUESTIONNAIRE

For village health worker

Dear village health worker,

We are asking many of the village health workers of communes that took part in the first use of Event-Based Surveillance (EBS) in Vietnam to tell us their views about the program. Village health workers are at the heart of system and your opinion is very valuable to us and will help to improve it for future roll-out in other areas of the country.

Please read these instructions before starting the survey.

This survey starts by asking a few questions about you, the person taking the survey. Please answer these as accurately as possible.

The second part of the survey contains 12 statements regarding different aspects of Event Based Surveillance that we would like to get your opinion on. In this section, you are asked to rate how strongly you agree or disagree with each statement, on a scale from 1 to 5 with 1 being the strong disagreement and 5 being the strong agreement.

The third part of the questionnaire asks a short series of questions which are answered by choosing “True” if you agree with the statement or “False” if you disagree.

For all questions, you can also choose the number 0, meaning you don’t know or don’t have an opinion.

The final section, asks for you to list a variety of possible things that might help us to improve future versions of the program.

Please read and answer all questions carefully.

Thank you very much for answering these questions and supporting Event-Based Surveillance in Vietnam.

SURVEY OF EBS ACCEPTABILITY. A SELF-ADMINISTERED QUESTIONNAIRE

For village health worker

General information

Commune ___________________________________ Date: _____________

Sex	Age (years)	Level of Education	Health care background
☐ Male	☐ less than 20 yr	☐ Less than primary school	☐ Yes
☐ Female	☐ 20-29 yr	☐ Primary school	☐ No
	☐ 30-39 yr	☐ Secondary school	☐ Don't know
	☐ 40-49 yr	☐ High school and equivalent	
	☐ 50 yr or older	☐ College and more	

Table #1. Please show your agreement or disagreement with the following sentences. Circle the number in the box that best shows the strength and type of your feelings.

	**With regard to having an event based surveillance system in my community, I feel that …**	**Strongly disagree**	**Somewhat Disagree**	**Neutral**	**Somewhat Agree**	**Strongly agree**	**Do not know**
1	As a result of the EBS training I now understand how to recognize signals.	1	2	3	4	5	0
2	As a result of the EBS training I now understand how to report signals into the EBS system	1	2	3	4	5	0
3	I received necessary support from the Commune Health Station to perform my duties of EBS.	1	2	3	4	5	0
4	The community members have been very helpful in reporting signals.	1	2	3	4	5	0
5	The EBS program is very important in the detection of public health events.	1	2	3	4	5	0
6	The EBS helps detect public health events earlier than before.	1	2	3	4	5	0
7	I have to do extra work within the current EBS system.	1	2	3	4	5	0
8	The EBS program should be continued in my commune.	1	2	3	4	5	0
9	I am willing to continue taking part in EBS program.	1	2	3	4	5	0
10	Additional training on EBS is needed.	1	2	3	4	5	0
11	Refresher trainings for EBS are needed.	1	2	3	4	5	0
12	Additional EBS guidelines are needed.	1	2	3	4	5	0

Table # 2. Please circle your responses to the questions. Also, in the last column, place a checkmark in your top three barriers to reporting.

	**Possible barriers to your participation in event-based surveillance**	**True**	**False**	**Do not have an opinion**	**Top barriers ✓**
13	I'm too busy to fulfill the duties required by EBS.	true	false	0	
14	I don't understand what to report.	true	false	0	
15	I don't know how to report.	true	false	0	
16	I don't know to whom I should report.	true	false	0	
17	I don't think EBS is useful.	true	false	0	
18	I don't have a strong interest in reporting.	true	false	0	
19	I don't get paid enough for the extra work EBS requires.	true	false	0	
20	The person to whom I'm supposed to report signals often cannot be reached.	true	false	0	
21	I feel that I did not receive enough training.	true	false	0	
22	If there are any barriers that you can think of that are not mentioned, list it below				

____

____

____

23. Please name 3 things that would make it easier for you to find and report signals that occur in your community:

    A.

    B.

    C.

24. What could the government do to better motivate you to report?

25. If you have additional comments or suggestions, please explain briefly:

Thank you very much for answering these questions.

Kindly seal the completed questionnaire in the envelope and return it to the Commune Health Station's EBS focal point by June XX

SURVEY OF EBS ACCEPTABILITY. A SELF-ADMINISTERED QUESTIONNAIRE

For EBS focal point at Commune Health Station (CHS)

Dear Commune Health Station (CHS)'s EBS focal point,

We are asking many of the focal points at Commune Health Stations that took part in the first use of Event-Based Surveillance (EBS) in Vietnam to tell us their views about the program. The focal points at Commune Health Stations are at the heart of system and your opinion is very valuable to us and will help to improve it for future roll-out in other areas of the country.

Please read these instructions before starting the survey.

This survey starts by asking a few questions about you, the person taking the survey. Please answer these as accurately as possible.

The second part of the survey contains 15 statements regarding different aspects of Event Based Surveillance that we would like to get your opinion on. In this section, you are asked to rate how strongly you agree or disagree with each statement, on a scale from 1 to 5 with 1 being the strong disagreement and 5 being the strong agreement.

The third part of the questionnaire asks a short series of questions which are answered by choosing "True" if you agree with the statement or "False" if you disagree.

For all questions, you can also choose the number 0, meaning you don't know or don't have an opinion.

The final section, asks for you to list a variety of possible things that might help us to improve future versions of the program.

Please read and answer all questions carefully.

Thank you very much for answering these questions and supporting Event-Based Surveillance in Vietnam.

SURVEY OF EBS ACCEPTABILITY. A SELF-ADMINISTERED QUESTIONNAIRE

For EBS focal point at Commune Health Station (CHS)

General information

Commune Health station (CHS):______________________________ Date: _____________

Sex	Age (years)	Level of Education
☐ Male	☐ less than 20 yr	☐ Less than primary school
☐ Female	☐ 20-29 yr	☐ Primary school
	☐ 30-39 yr	☐ Secondary school
	☐ 40-49 yr	☐ High school and equivalent
	☐ 50 yr or older	☐ College and more

Table # 1. Please show your agreement or disagreement with the following sentences. Circle the number in the box that best shows the strength and type of your feelings.

	**With regard to having an event based surveillance system in my community, I feel that …**	**Strongly disagree**	**Somewhat Disagree**	**Neutral**	**Somewhat Agree**	**Strongly agree**	**Do not know**
1	As a result of the EBS training I now understand how to do triage of signals.	1	2	3	4	5	0
2	As a result of the EBS training I now understand how to report signals to the district	1	2	3	4	5	0
3	I received necessary support from the District Preventive Medical Center to perform my duties of EBS.	1	2	3	4	5	0
4	The village health volunteers have been proactive in reporting signals.	1	2	3	4	5	0
5	The EBS program is very important in the detection of public health events.	1	2	3	4	5	0
6	The EBS helps detect public health events earlier than before.	1	2	3	4	5	0
7	I have to do extra work within the current EBS system.	1	2	3	4	5	0
8	The EBS program should be continued in my commune.	1	2	3	4	5	0
9	I am willing to continue taking part in EBS program.	1	2	3	4	5	0
10	An electronic version of log books would be useful for reporting signals.	1	2	3	4	5	0
11	The log books are easy to understand.	1	2	3	4	5	0
12	The logbooks are easy to complete.	1	2	3	4	5	0

	Continuation… **With regard to having an event based surveillance system in my community, I feel that …**	**Strongly disagree**	**Somewhat Disagree**	**Neutral**	**Somewhat Agree**	**Strongly agree**	**Do not know**
13	Additional training on EBS is needed.	1	2	3	4	5	0
14	Refresher trainings for EBS are needed.	1	2	3	4	5	0
15	Additional EBS guidelines are needed.	1	2	3	4	5	0

Table # 2. Please circle your responses to the questions. Also, in the last column, place a checkmark in your top three barriers to reporting.

	**Possible barriers to your participation in event-based surveillance**	**True**	**False**	**Do not have an opinion**	**Top barriers ✓**
16	I'm too busy to fulfill the duties required by EBS.	true	false	0	
17	I don't understand how to do triage.	true	false	0	
18	I don't know to whom I should report signals.	true	false	0	
19	I don't know how to accurately summarize EBS data and send the summary reports to the next level up.	true	false	0	
20	I don't think EBS is useful.	true	false	0	
21	I don't have a strong interest in reporting.	true	false	0	
22	I don't get paid enough for the extra work EBS requires.	true	false	0	
23	The person to whom I'm supposed to report signals often cannot be reached.	true	false	0	
24	I feel that I did not receive enough training.	true	false	0	
25	If there are any barriers that you can think of that are not mentioned, list it below:				

____

____

____

26. In your experience, which of the following have been detecting and reporting signals in your community during the past 4 weeks? *Check all that apply*

Community member……...☐	Village health workers...☐	Healers…………...☐
Teacher or School Staff…..☐	Private-owned clinic…. ☐	Religious leader.....☐
Office/Factory……………☐	Pharmacy……………...☐	Media…………....☐
Others……………………..☐	Specify: __________________	

27. Please name 3 things that would make it easier for you to do triage of signals.

A.

B.

C.

28. What could the government do to better motivate you?

29. If you have additional comments or suggestions, please explain briefly:

Thank you very much for answering these questions.

Kindly seal the completed questionnaire in the envelope and return it to the district's EBS focal point by June XX

SURVEY OF EBS ACCEPTABILITY. A SELF-ADMINISTERED QUESTIONNAIRE
For EBS focal point at district

Dear District's EBS focal point,

We are asking to all focal points at districts that took part in the first use of Event-Based Surveillance (EBS) in Vietnam to tell us their views about the program. The focal points at districts are at the heart of system and your opinion is very valuable to us and will help to improve it for future roll-out in other areas of the country.

Please read these instructions before starting the survey.

This survey starts by asking a few questions about you, the person taking the survey. Please answer these as accurately as possible.

The second part of the survey contains 15 statements regarding different aspects of Event Based Surveillance that we would like to get your opinion on. In this section, you are asked to rate how strongly you agree or disagree with each statement, on a scale from 1 to 5 with 1 being the strong disagreement and 5 being the strong agreement.

The third part of the questionnaire asks a short series of questions which are answered by choosing "True" if you agree with the statement or "False" if you disagree.

For all questions, you can also choose the number 0, meaning you don't know or don't have an opinion.

The final section, asks for you to list a variety of possible things that might help us to improve future versions of the program.

Please read and answer all questions carefully.

Thank you very much for answering these questions and supporting Event-Based Surveillance in Vietnam.

SURVEY OF EBS ACCEPTABILITY. A SELF-ADMINISTERED QUESTIONNAIRE

For EBS focal point at district

General information

District: ______________________________ Date: __________

Sex	Age (years)	Level of Education
☐ Male	☐ less than 20 yr	☐ Less than primary school
☐ Female	☐ 20-29 yr	☐ Primary school
	☐ 30-39 yr	☐ Secondary school
	☐ 40-49 yr	☐ High school and equivalent
	☐ 50 yr or older	☐ College and more

Table # 1. Please show your agreement or disagreement with the following sentences. Circle the number in the box that best shows the strength and type of your feelings.

	**With regard to having an event based surveillance system in my district, I feel that …**	**Strongly disagree**	**Somewhat Disagree**	**Neutral**	**Somewhat Agree**	**Strongly agree**	**Do not know**
1	As a result of the EBS training I now understand how to verify signals.	1	2	3	4	5	0
2	As a result of the EBS training I now understand how to report events to the PPMC.	1	2	3	4	5	0
3	I received necessary support from the Provincial Preventive Medicine Center (PPMC) to perform my duties of EBS.	1	2	3	4	5	0
4	The Commune Health Stations have been proactive in reporting signals.	1	2	3	4	5	0
5	The EBS program is very important in the detection of public health events.	1	2	3	4	5	0
6	The EBS helps detect public health events earlier than before.	1	2	3	4	5	0
7	I have to do extra work within the current EBS system.	1	2	3	4	5	0
8	The EBS program should be continued in my district.	1	2	3	4	5	0
9	I am willing to continue taking part in EBS program.	1	2	3	4	5	0
10	An electronic version of log books would be useful for reporting events.	1	2	3	4	5	0
11	The log books are easy to understand.	1	2	3	4	5	0

	Continuation… **With regard to having an event based surveillance system in my community, I feel that …**	**Strongly disagree**	**Somewhat Disagree**	**Neutral**	**Somewhat Agree**	**Strongly agree**	**Do not know**
12	The log books are easy to complete.	1	2	3	4	5	0
13	Additional training on EBS is needed.	1	2	3	4	5	0
14	Refresher trainings for EBS are needed.	1	2	3	4	5	0
15	Additional EBS guidelines are needed.	1	2	3	4	5	0

Table # 2. Please circle your responses to the questions. Also, in the last column, place a checkmark in your top three barriers to reporting.

	**Possible barriers to your participation in event-based surveillance**	**True**	**False**	**Do not have an opinion**	**Top barriers ✓**
16	I'm too busy to fulfill the duties required by EBS.	true	false	0	
17	I don't understand how to verify signals.	true	false	0	
18	I don't know to whom I should report events.	true	false	0	
19	I don't know how to accurately summarize EBS data and send the summary reports to the next level up.	true	false	0	
20	I don't think EBS is useful.	true	false	0	
21	I don't have a strong interest in verifying signals.	true	false	0	
22	I don't get paid enough for the extra work EBS requires.	true	false	0	
23	The person to whom I'm supposed to report events often cannot be reached.	true	false	0	
24	I feel that I did not receive enough training.	true	false	0	
25	If there are any barriers that you can think of that are not mentioned, list it below:				

____

____

____

26. Please name 3 things that would make it easier for you to verify signals and report events that occur in your district:

    A.

    B.

    C.

27. What could the government do to better motivate you?

28. If you have additional comments or suggestions, please explain briefly:

Thank you very much for answering these questions.

Kindly seal the completed questionnaire in the envelope and return it to the PPMC's EBS focal point by June XX

SURVEY OF EBS ACCEPTABILITY. A SELF-ADMINISTERED QUESTIONNAIRE

For EBS focal point at Province

Dear Provincial's EBS focal point,

We are asking the focal points at provinces that took part in the first use of Event-Based Surveillance (EBS) in Vietnam to tell us their views about the program. The focal points at provinces are at the heart of system and your opinion is very valuable to us and will help to improve it for future roll-out in other areas of the country.

Please read these instructions before starting the survey.

This survey starts by asking a few questions about you, the person taking the survey. Please answer these as accurately as possible.

The second part of the survey contains 10 statements regarding different aspects of Event Based Surveillance that we would like to get your opinion on. In this section, you are asked to rate how strongly you agree or disagree with each statement, on a scale from 1 to 5 with 1 being the strong disagreement and 5 being the strong agreement.

The third part of the questionnaire asks a short series of questions which are answered by choosing "True" if you agree with the statement or "False" if you disagree.

For all questions, you can also choose the number 0, meaning you don't know or don't have an opinion.

The final section, asks for you to list a variety of possible things that might help us to improve future versions of the program.

Please read and answer all questions carefully.

Thank you very much for answering these questions and supporting Event-Based Surveillance in Vietnam.

SURVEY OF EBS ACCEPTABILITY. A SELF-ADMINISTERED QUESTIONNAIRE
For EBS focal point at province

General information

Province: ______________________________ Date: ____________

Sex: ☐ Male ☐ Female

Age (years): ☐ less than 20 yr ☐ 20-29 yr ☐ 30-39 yr ☐ 40-49 yr ☐ 50 yr or older

---

Table # 1. Please show your agreement or disagreement with the following sentences. Circle the number in the box that best shows the strength and type of your feelings.

	**With regard to having an event based surveillance system in my province, I feel that …**	**Strongly disagree**	**Somewhat Disagree**	**Neutral**	**Somewhat Agree**	**Strongly agree**	**Do not know**
1	As a result of the EBS training I now understand how to respond to events.	1	2	3	4	5	0
2	I received necessary support from the Regional Institute (RI) to perform my duties of EBS.	1	2	3	4	5	0
3	The districts have been proactive in reporting events.	1	2	3	4	5	0
4	The EBS program is very important in the detection of public health events.	1	2	3	4	5	0
5	The EBS helps detect public health events earlier than before.	1	2	3	4	5	0
6	I have to do extra work within the current EBS system.	1	2	3	4	5	0
7	The EBS program should be continued in my province.	1	2	3	4	5	0
8	I am willing to continue taking part in EBS program.	1	2	3	4	5	0
9	An electronic version of log books would be useful for reporting up.	1	2	3	4	5	0
10	The log books are easy to understand.	1	2	3	4	5	0
11	The log books are easy to complete.	1	2	3	4	5	0
12	Additional training on EBS is needed.	1	2	3	4	5	0
13	Refresher trainings for EBS are needed.	1	2	3	4	5	0
14	Additional EBS guidelines are needed.	1	2	3	4	5	0

Table # 2. Please circle your responses to the questions. Also, in the last column, place a checkmark in your top three barriers to reporting.

	**Possible barriers to your participation in event-based surveillance**	**True**	**False**	**Do not have an opinion**	**Top barriers ✓**
15	I'm too busy to fulfill the duties required by EBS.	true	false	0	
16	I don't know how to respond to an event.	true	false	0	
17	I don't know to whom I should report events.	true	false	0	
18	I don't know how to accurately summarize EBS data and send the summary reports to the next level up.	true	false	0	
19	I don't think EBS is useful.	true	false	0	
20	I don't have a strong interest in responding to events	true	false	0	
21	I don't get paid enough for the extra work EBS requires.	true	false	0	
22	The person to whom I'm supposed to report events often cannot be reached.	true	false	0	
23	I feel that I did not receive enough training.	true	false	0	
24	If there are any barriers that you can think of that are not mentioned, list it below:				

____

____

____

25. Please name 3 things that would make it easier for you to respond to events that occur in your province:

    A.

    B.

    C.

26. If you have additional comments or suggestions, please explain briefly:

Thank you very much for answering these questions.

Kindly seal the completed questionnaire in the envelope and return it to the RI's EBS focal point by June XX
